# The future of cancer treatment: combining radiotherapy with immunotherapy

**DOI:** 10.3389/fmolb.2024.1409300

**Published:** 2024-07-09

**Authors:** Gunjan Dagar, Ashna Gupta, Abhishek Shankar, Ravi Chauhan, Muzafar A. Macha, Ajaz A. Bhat, Dayasagar Das, Rajeev Goyal, Sandeep Bhoriwal, Raj K. Pandita, Chandra Prakash Prasad, Partha S. Sarkar, Tej K. Pandita, Mayank Singh

**Affiliations:** ^1^ Department of Medical Oncology, All India Institute of Medical Sciences, New Delhi, India; ^2^ Department of Radiation Oncology, All India Institute of Medical Sciences, New Delhi, India; ^3^ Watson-Crick Centre for Molecular Medicine, Islamic University of Science and Technology, Pulwama, Jammu And Kashmir, India; ^4^ Department of Human Genetics-Precision Medicine in Diabetes, Obesity and Cancer Program, Sidra Medicine, Doha, Qatar; ^5^ Department of Medicine, NYU Langone Health, New York City, NY, United States; ^6^ Department of Biochemistry, Lady Harding Medical College, New Delhi, India; ^7^ Department of Surgical Oncology, All India Institute of Medical Sciences (AIIMS), New Delhi, India; ^8^ Center for Genomics and Precision Medicine, Texas A and M College of Medicine, Houston, TX, United States; ^9^ Department of Neurobiology and Department of Neurology, University of Texas Medical Branch, Galveston, TX, United States

**Keywords:** immunotherapy, radiotherapy, cancer, metastasis immunotherapy, metastasis, cancer therapy, DNA Damage, DNA damage response

## Abstract

Radiotherapy (RT) and immunotherapy (IT) are the powerful tools for cancer treatment which act through the stimulation of immune response, and evidence suggest that combinatorial actions of these therapies may augment each other’s beneficial effect through complex synergistic mechanisms. These molecular strategies are designed to target rapidly dividing cancer cells by either directly or indirectly inducing DNA damage. However, when cells detect DNA damage, they activate a range of signalling pathways known as the DNA damage response (DDR) to repair. Strategies are being developed to interfere with the DDR pathways in cancer cells to ensure their damage-induced degeneration. The stability of a cell’s genetic material is largely dependent on the efficacy of DNA repair and therefore, an in-depth understanding of DNA damages and repair mechanism(s) in cancer cells is important to develop a promising therapeutic strategies for ensuring the efficacy of damage-induced tumor cell death. In recent years, a wide range of small molecule drugs have been developed which are currently being employed to combat the DNA repair deficiencies associated with tumor cells. Sequential or concurrent use of these two modalities significantly enhances the anti-tumor response, however with a concurrent probability of increased incidence of symptomatic adverse effects. With advent of newer IT agents, and administration of higher doses of radiation per fraction, such effects are more difficult to predict owing to the paucity of randomized trial data. It is well established that anti cytotoxic-T-lymphocyte-associated antigen 4 (CTLA-4), anti- Programmed cell death protein 1(PD-1), anti-Programmed cell death one ligand 1 (PD-L1) can be safely administered with RT and many studies have demonstrated survival benefit with such combination for patients with metastatic malignancy. However, the biology of radioimmunotherapy (RT/IT) is still an open area where research need to be focused to determine optimum dosage specially the interaction of the RT/IT pathways to determine optimum dosing schedule. In the current article we have summarised the possible intracellular immunological events that might be triggered when RT and IT modalities are combined with the DDR antagonists and highlighted present clinical practices, outcome, and toxicity profile of this novel treatment strategy.

## Introduction

Anticancer treatment consists of an armamentarium of many modalities, like surgery, chemotherapy (CT), radiotherapy (RT) and immunotherapy (IT). In the last decade, treatment with ITs has emerged as an extremely power tool for the treatment of cancer. Immunotherapy can deliver personalised treatment as per the oncogenic profile of a specific target in various solid malignancies. The human immune system can adapt dynamically to keep pace with the rate of mutation and growth of cancer cells (Tang et al., 2014). The immune system has an innate ability to “remember” cancer cells; therefore IT, can offer targeted treatment and protection against cancer recurrence. The majority of targeted drugs exhibit restricted effectiveness against solid tumors, primarily attributed to the frequent development of resistance to these treatments (Vaddepally et al., 2020). In recent times, IT such as adoptive cell treatment and immunological checkpoint blockade (ICB), has demonstrated impressive clinical effectiveness. The use of ICBs to treat solid tumors has been authorised for cancer treatment targeting a wide range of molecules, including CTLA4, PD1, and ligand 1 (PDL1) (Zhu et al., 2021a). Anti-PD1 therapy has emerged as a leading form of ICB therapy, outperforming the anti-CTLA4 therapy in various tumor types. However, its efficacy as a stand-alone treatment is typically limited, with a response rate of only about 20% particularly for the advanced stage cancers (Kim et al., 2022). In addition, cancer cells often develop adaptive immune resistance mechanisms to evade immune system attacks. Given these challenges, combining IT with complementary approaches is a rational strategy to enhance the anti-tumor effects (Zimmer et al., 2020; Zhu et al., 2021b).

A key component of cancer care is RT in combination with surgery and systemic therapies such as IT, CT, and targeted therapies. The primary goal of RT is to enhance the delivery of radiation to the tumor, thereby ensuring local control while minimising radiation exposure to the adjacent healthy tissue. Advancements like High Linear Energy Transfer (LET) or Intensity-Modulated RT (IMRT) have significantly improved the therapeutic ratio (Elshaikh et al., 2006). IMRT indeed represents a significant advancement in RT techniques. Its ability to modulate the intensity of radiation beams allows for more precise targeting of tumors while sparing the surrounding healthy tissues. This precision translates into better tumor coverage and reduced toxicity compared to the conventional RT. IMRT achieves this by dividing the radiation beam into many smaller beamlets, each with varying intensity levels. By adjusting the intensity of these beamlets and their angles, radiologists can tailor the radiation dose to conform closely to the tumor area, delivering higher doses to the tumor while minimising the radiation exposure to the nearby normal tissues (Eisbruch et al., 1998). However, despite these significant advances, many patients still encounter local recurrences of the disease following RT. Most of the DNA damage, particularly the double-strand breaks (DSBs), is what causes the RT-induced cell death. As a result, tumor cells with effective DNA repair systems are resistant to ionizing radiation, but tumor cells with impaired DSB repair pathways are more susceptible to death (Zhu et al., 2021a). Therefore, treatments of the tumor cells with small molecule inhibitors that block or impair the machinery responsible for repairing DNA damage may improve the overall effectiveness of RT.

CT, another prevalent form of genotoxic treatment for cancer therapy, employs a class of pharmaceutical small molecule agents that induce DNA damage through various mechanisms, including topoisomerase inhibition, DNA alkylation, and crosslinking (Elshaikh et al., 2006). Often administered alongside RT or surgery, CT has been demonstrated to influence the host immune response. Both CT and RT have been shown to interact with the immune checkpoint inhibitors (ICIs) in a synergistic manner.

The DNA damage response (DDR) plays a crucial role in preserving the genomic stability through the restoration of various forms of DNA damage in cells (Jeggo and Lavin, 2009). Compared to normal cells, cancer cells with high underlying levels of DNA damage and genomic instability depend more on DDR for the survival **(**DNA damage accumulates because of DDR deficiencies, which also increase tumor immunogenicity). Malfunctions of the DDR pathway can arise from mutation(s) in various genes involved in DDR pathway and/or due to epigenetic modifications of the DDR proteins that result in the impaired and/or dysfunctional DDR (Chabner and Roberts, 2005). Therefore, combining CT, IR therapy, and IT with DDR network inhibitors has been drawing increasing attention in a large number of clinical trials.

In this review, we will explore various aspects, including different types of DNA damage and their repair pathways, the synergy of DDR with various cancer treatment options such as RT, CT, and IT.

## DNA damage response in eukaryotes

All organisms must maintain the DNA sequence integrity and fidelity of their genome to survive and function properly. The eukaryotic genome faces continuous challenges from a wide variety of external and internal sources of DNA damage, including reactive oxygen species (ROS), IR, UV light and various exogenous chemical agents that can induce different types of damages in genome (Das et al., 2023). To address the fundamental issue of genomic erosion, organisms have developed an intricate network of the DDR systems. This network encompasses various damage tolerance processes, DNA repair mechanisms, and cell-cycle checkpoint pathways. The primary regulators of DDR signalling pathway that are activated via phosphorylation in response to DNA damage is the Ataxia-telangiectasia mutated (ATM) and Ataxia-telangiectasia and Rad3 related (ATR) kinases. ATM, a serine-threonine kinase serves as the primary orchestrator in the cellular response to DNA DSBs induced by exposure to IR and stalled replication forks. On the other hand, ATR plays a major role in single-strand DNA damage repair mechanism (Jurkovicova et al., 2022). Two extensively studied downstream targets of ATM and ATR are the cell cycle checkpoint kinases CHK1 and CHK2. The coordinated action of these proteins initiate the ATM and/or ATR kinase-initiated signalling cascade and play critical roles in determining the cellular responses to DNA damage (Dagar et al., 2023). The activated CHK1/CHK2 kinases further phosphorylate p53 and CDC25. The activation of this phosphorylation cascade results in increased expression of p21 (regulated by p53), and inhibition of CDK activity, which ultimately leads to cell cycle arrest at the G1-S and G2-M transitions (regulated by CDC25 and WEE1) (Mushtaq et al., 2021).

The molecular pathways of DNA repair mechanisms involved in addressing common types of DNA damage are the nucleotide excision repair (NER), homologous recombination (HR), non-homologous end joining (NHEJ), base excision repair (BER), and mismatch repair (MMR). These DNA damage repair pathways play crucial roles in maintaining the genome integrity of mammalian cells.

Moreover simple solution that has evolved in response to DNA damage involves the direct reversal of the DNA lesions through the activities of specialized enzymes. Examples include photolyases, which selectively reverse the UV-induced DNA damage (Jurkovicova et al., 2022), and the suicide enzyme O6-methylguanine transferase (MGMT), which is involved in repairing specific types of DNA lesions. It operates by repairing damaged guanine nucleotides and transferring the methyl group at the guanine’s O6 site to its cysteine residues. This enzymatic activity helps prevent gene mutations, cell death, and the onset of tumorigenesis induced by alkylating agents (Groelly et al., 2023). Because the photolyases are not conserved in mammals, they heavily rely on intricate molecular processes for the repair and elimination of the UV-induced damages viz. NER.

Nucleotide excision repair eliminates a diverse range of single-strand lesions that cause local helix destabilization. NER is a multifaceted, intricate multistep process that requires the coordinated action of around 25 distinct polypeptides and plays a pivotal role in eliminating bulky DNA intra-strand and interstrand crosslinks (ICL) adducts (Weber, 2005). NER plays a pivotal role in eliminating bulky DNA adducts on DNA and contributes to the repair of intra-strand and inter-strand crosslinks (ICLs). The xeroderma pigmentosum (XP) proteins, along with excision repair cross-complementation group 1 (ERCC1), both play essential roles in the NER pathway. NER is involved in two types of repairs: global genome NER (GG-NER) and transcription-coupled NER (TC-NER). If the damage occurs within the actively transcribed strands of genes, TC-NER mechanism is activated. In the TC-NER mechanism, the detection of DNA damage in the transcribing DNA is carried out by the stalled RNA polymerase. The Cockayne syndrome factors A and B (CSA and CSB) play essential roles in orchestrating the formation and function of the TC-NER complex (NCBI, 2024b). If damage is not in the actively transcribed strand of a gene, then GG-NER is initiated. A dimer consisting of XPC and HR23B appears to recognize and bind to the damaged DNA. This is followed by the binding of the general transcription factor TFIIH and XPA, a DNA binding protein. The binding of XPA facilitates the binding of Replication Protein A (RPA). This heterotrimer complex stabilizes the unwound DNA and guides the two structure-specific endonucleases, the ERCC1-XPF complex and XGP. Excision of damaged DNA is followed by the replicative gap-repair proteins that carry out DNA synthesis, the final nick is sealed by DNA ligase I (Hoeijmakers, 1993).

Lesions that serve as substrates for both NER and BER are situated within one of the DNA strands. Some genotoxic agents, such as IR, and various chemotherapeutic drugs, affect both the strands of DNA and induce DSBs which are repaired by two distinctly different pathways: NHEJ and HR (Groelly et al., 2023). HR is characterized by its high fidelity of DNA repair. The process initiates with the recognition and processing of the damaged DNA by the CtlP and MRN (MRE11-RAD50-NBS1) complex through end resection, enabling the binding of RPA to the single-stranded overhang. The PALB2, BRCA1, and BRCA2, and complex recruit RAD51, which displaces RPA. Through strand invasion, the processed DNA filament attaches to the intact DNA to create the new complementary DNA strand (Fousteri and Mullenders, 2008).

The NHEJ mechanism stands out from the HR as it does not rely on template DNA for the repair process as NHEJ is an error-prone repair mechanism. NHEJ breaks are recognized by Ku heterodimer (Ku70/80) subunits that activate DNA-PK, a PI3-kinase. XRCC4 and DNA ligase IV are also recruited to the DSB sites to ligate the DNA ends and fill in any gaps in the sequence. Later, XRCC4, Pol μ, and DNA Ligase four are then recruited to the DSB sites and ligate the DNA ends together (Giglia-Mari et al., 2011). In the absence of essential NHEJ components, the Alternative End Joining (A-EJ) pathway, also referred to as microhomology-mediated end joining, becomes more prominent in response to DDR. A-EJ relies on PARP1 and DNA polymerase theta (Pol θ), to facilitate the re-joining of two DNA ends, utilizing very short homologous sequences typically ranging from 2 to 20 base pairs (San Filippo et al., 2008). Decreased expression or loss of certain NHEJ proteins, such as Rev7 and 53BP1, can result in resistance to PARP inhibitors, particularly in BRCA1-deficient cancers**.**

The BER pathway corrects minor base lesions that do not significantly disrupt the DNA double-helix structure. The key elements of the repair pathway include DNA polymerases, endonucleases, glycosylases, and DNA ligases. PARP1 and PARP2 help to facilitate the process (Burma et al., 2006). Damage bases are first identified and removed by DNA glycosylases creating apurinic or apyrimidinic (AP) sites. Both PARP1 and apurinic/apyrimidinic endonuclease 1 (APE1) can detect and bind the damage sites. This triggers the catalysis of poly ADP-ribosylation (PAR) on various protein substrates, facilitating the recruitment of repair proteins to the site of damage. The subsequent synthesis and ligation step of BER is bifurcated into two sub-pathways—short-patch and long-patch (Ceccaldi et al., 2015). In short-patch BER, the gap is filled with the correct base pair by polymerase beta (Pol β). The consecutive ligation of DNA ends requires either the DNA ligase I (LIG1) or the complex formed by X-ray repair cross-complementing protein 1 (XRCC1) and DNA ligase III (LIG3). While in long-patch BER, the process involves lap endonuclease-1 (FEN1), proliferating cell nuclear antigen (PCNA) (NCBI, 2024c) polymerase delta/epsilon (Pol δ/ε), replication factor-C (RFC), and LIG1 (Barakat et al., 2012)**.**

Preserving the genome stability is largely dependent on MMR system, a biological pathway that is highly conserved. Base-base mismatches and insertion/deletion mismatches produced during DNA replication and recombination are the main sources of MMR’s specificity (Maynard et al., 2009). Additionally, MMR inhibits HR and has been implicated in the signalling of DNA damage in eukaryotic cells. An MSH/MSH6 (MutSa) or MSH2/MSH3 (MutSb) ATPase heterodimer is assembled to produce a sliding clamp that is responsible for detecting and initiating the DNA repair (Li, 2008). Other elements are brought to the site after the mismatch is identified. With endonuclease activity, the MLH1/PMS2 (MutLa) complex makes a first cut in the helix, which is followed by a larger EXO1-mediated resection. MSH2-MSH3 detects longer lengths of mismatches, while the MSH2-MSH6 complex detects shorter ones. MLH1- PMS2, PMS2, and EXO1 are involved in excising the mismatched DNA (Strzalka and Ziemienowicz, 2011).

## IR-induced DNA damage in cancer therapy

**FIGURE 1:**
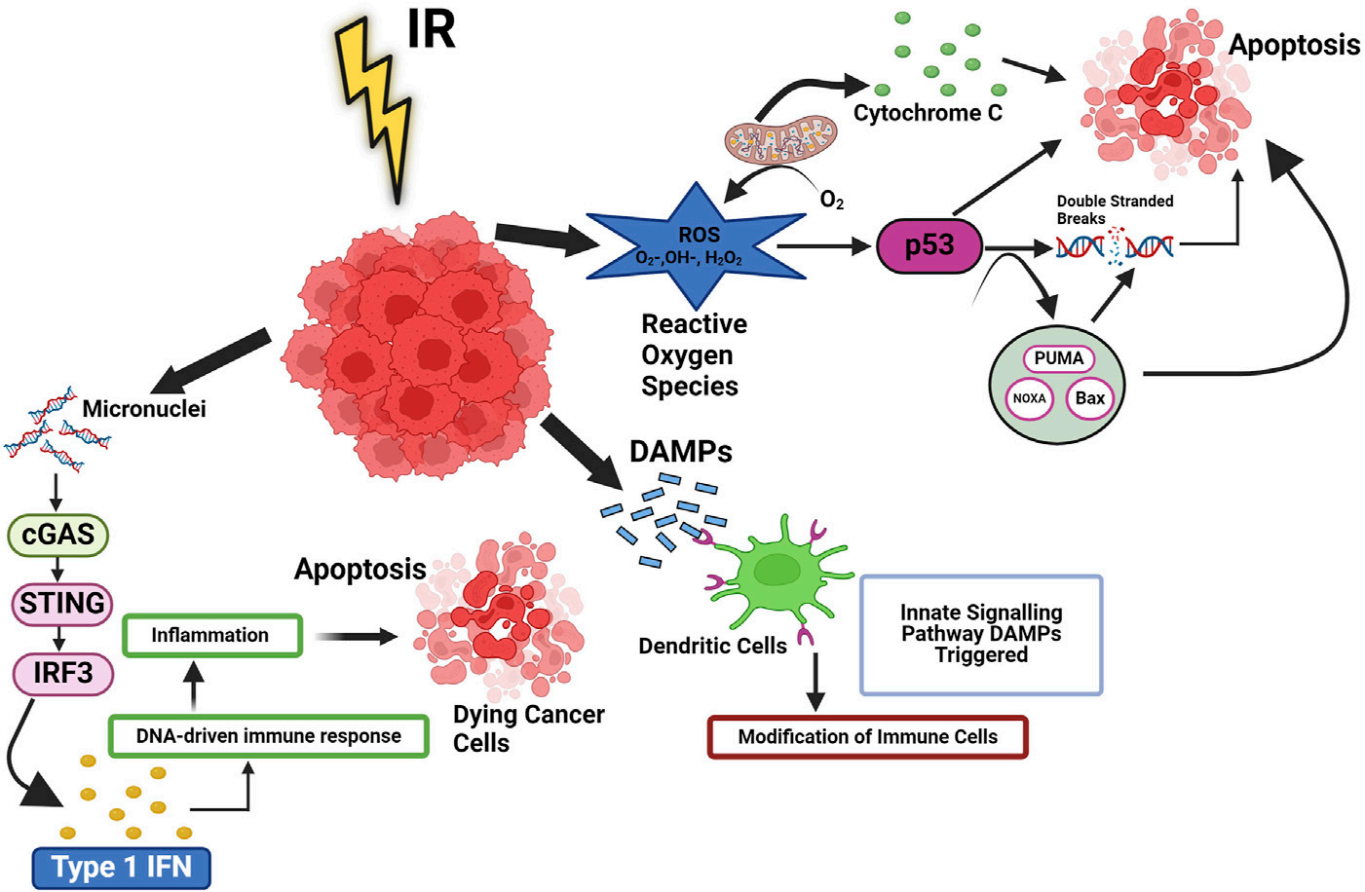
Ionizing radiation induced cellular pathways in cancer. Ionizing radiation (IR) trigger cell death, which leads to release of DAMPs and cytokines, then activate innate signalling pathways and modification of immune cells. These signals improve the processing of TAAs and the cross-presentation of antigenic peptides to CD8^+^ T lymphocytes via MHC I. They also favour the recruitment of APCs like DCs, the uptake of dying tumor cells, and the processing of TAAs. Additionally, radiation can cause the release of type-I IFN from immunological and cancer cells as well as activate the complement system, which can increase the activation of both DC and T cells. RT can also result in MHC I upregulation. p53 signalling is linked to the reactive oxygen species (ROS) response to radiation. Radiation-induced mitochondrial damage helps irradiation boost intracellular ROS levels. When ROS levels are high, p53 may significantly reduce the oxidative damage imposed on by radiation. Type I IFN is produced when cytoplasmic chromatin DNA stimulates the cGAS-STING pathway and regulates DNA derived immune response.

IR is a cornerstone in cancer therapy, leveraging its ability to induce DNA damage to eradicate malignant cells. Therefore, understanding the biochemical and molecular basis by which IR induce DNA damage can provide useful information. Understanding these mechanisms is crucial for comprehending the genotoxic effects of IR. There are some primary mechanisms of IR-induced DNA damage, such as direct ionization which directly interacts with the DNA molecule, leading to ionization. It causes the ejection of electrons from atoms within the DNA molecule, resulting in the formation of ion pairs and free radicals, or an indirect effect through water radiolysis. In this mechanism, IR, indirectly induce DNA damage by ionizing water molecules in the cellular environment through a process known as radiolysis (Fortini and Dogliotti, 2007). Radiolysis of water produces ROS, such as hydroxyl radicals (•OH), hydrogen peroxide (H_2_O_2_), and superoxide radicals (O2•−). These ROS can then interact with DNA and cause damage (Barakat et al., 2012) (Figure 1).

IR directly causes DSBs while also causes base damage through indirect effects. Moreover, IR also leads to the formation of ROS, which indirectly contributes to DNA damage. These ROS cause DNA to develop apurinic/apyrimidinic (abasic) sites, SSBs, changes to the sugar moiety, and deaminated adducted bases (Srinivas et al., 2018). When DNA sustains damage, the cell’s repair machinery is activated, prompting the halting of the cell cycle at specific control checkpoints. This pause allows the cell to undertake the repair of the DNA damage, thereby preventing the cell cycle from progressing further.

ROS can induce various types of DNA damages, including generation of abasic sites, SSBs, chemical modifications to the sugar moiety, and deaminated adducted bases. When DNA damage occurs, the cell activates repair mechanisms and halts the cell cycle at specific checkpoints to facilitate DNA repair. This response is crucial for preventing the propagation of mutations and maintaining the genomic stability (Davalli et al., 2018).

In the context of RT, if tumor cells possess efficient DNA repair mechanisms, they can develop resistance to radiation by repairing the damage induced by IR. This resistance allows tumor cells to survive and continue replicating despite the radiation treatment. However, if the DNA damage remains unrepaired or is too severe, the cell may undergo programmed cell death, known as apoptosis, to prevent the transmission of mutations to daughter cells (Curtin, 2023).

High doses of radiation can lead to toxicity and diminish the patient’s prognosis. Therefore, tailoring radiation treatment based on the individual’s DSB repair capability may help predict the toxicity to surrounding tissues, thus enhancing treatment safety (NCBI, 2024a). The DNA repair capacity holds important significance in determining the suitable treatment strategy for cancer patients, and functional tests can offer valuable insights for making these clinical decisions.

It is well known that IR directly induces DNA damage in cancer cells, which leads to the activation of systemic IR-induced signalling cascade leading to changes in tumor microenvironment, These changes tend to influence the tumor microenvironment and make the tumors much more receptive to IT by aiding the release of tumor antigens, which can be targeted by IT (Sharabi et al., 2015) by increasing the density of TILs (Tumor-infiltrating lymphocytes). It has also been shown that IR leads to cyclic GMP-AMP synthase (cGAS) activation, a stimulator of the interferon gene (STING) pathway, leading to the production of proinflammatory signals (McLaughlin et al., 2020) that have a bearing on the fate of cancer cells and tumors. However, it is important to note that there have been few reports that suggest that IR gives rise to immune suppression as well by modulating myeloid-derived suppressor cells (MDSCs) and regulatory T cells (Tregs) (Rodríguez-Ruiz et al., 2018). In the irradiated tumor microenvironment, several intracellular signalling pathways are also modulated by IR in cancer cells. Radiation induces the death of cancer cells primarily by inducing DSBs and the generation of ROS, as shown in Figure 1, which modulate the intracellular tumor microenvironment (Dagar et al., 2023). The formation of micronuclei is a phenomenon that occurs upon exposure to IR, which further leads to activation of the cGAS-STING pathway, leading to the production of type 1 interferon (IFN**).** In addition to nuclear DNA, mitochondrial DNA damage is also recognized by cGAS, which then forms a complex with DNA (NCBI, 2023). This leads to the formation of the second messenger cGAMP, which modulates downstream signalling for the production of type 1 Interferons, leading to the maturation of dendritic cells (DCs), as observed by an increase in 1) the expression of co-stimulatory molecules and 2) the migratory capacity of DCs (Sprooten et al., 2019). The fragmented cancer cells are exposed to IR release factors like damage-associated molecular patterns (DAMPs), which influence the functioning of different immune cells (Figure 1). NF-κB pathways (canonical and non-canonical) also play an important role in IR-induced responses. Beside cGAS-STING pathways, the NF-κB pathway is commonly mediated through the IKK dependent canonical pathway. Which works with IRF-3 to optimise the expression of the IFN-β gene (Garland et al., 2022). The association of IRF3 and NF-κB is essential for activating type I IFN in DCs, which are stimulated by irradiated tumor.

Although RT-induced DSBs are the most effective molecular events for damaging and killing cancer cells, the DNA damage repair capabilities inherent in cancer cells can lead to resistance and diminish the efficacy of therapy. This radiation induces various types of DNA damages, including complex DSBs, which are pivotal in eliminating tumor cells. RT employs fractionated doses of IR to exploit this property, generating significant levels of clustered DNA damage that challenge tumor cells’ repair mechanisms and impede their survival chances.

Additionally, the effectiveness of RT is augmented by adjuncts that enhances the sensitivity of hypoxic cells, which are often resistant to radiation treatment. These adjuncts capitalise on vulnerabilities within tumor cells’ DNA repair pathways, such as deficiencies in repair pathways like HR due to mutations in BRCA1 resulting in repair deficiency. Tumor cells with compromised repair mechanisms are more susceptible to the DNA damage induced by RT, leading to their targeted destruction (Deckbar et al., 2011). However, it is crucial to consider the potential impact of lower radiation doses on normal tissues. While tumor cells may receive cytotoxic doses, normal cells may experience non-DSB clustered DNA damage even at lower radiation doses. For instance, doses as low as 10–100 cGy have been observed to induce clusters of apurinic/apyrimidinic (AP) sites in primary human fibroblasts (Guo et al., 2011). This emphasises the necessity of striking a balance between the therapeutic benefits of RT for the cancer cells and the potential risks to the surrounding normal tissues. The treatment planning in RT aims to maximise the radiation dose to tumor cells while minimizing possible exposure to the surrounding healthy tissues, thereby reducing the likelihood of adverse effects. Ongoing research endeavours to refine RT techniques and develop adjunct therapies to enhance tumor cell eradication while safeguarding normal tissues from radiation-induced harm (Guo et al., 2011). Therefore, focusing on inhibiting DNA damage repair mechanisms, such as by inhibiting NHEJ or by targeting single-strand break repair (SSBR) and BER pathway, presents a promising therapeutic approach to enhance the sensitivity of cancer cells to RT, offering a precise and effective treatment strategy for cancer patients. The SSBR and BER pathways are responsible for repairing damaged bases and SSBs in DNA. Inhibiting BER/SSBR could result in unrepaired damage, which may convert to DSBs when encountering a replication fork. Thus, in cells that are already HR-deficient, like breast or ovarian cancer tumors (BRCA^−/−^), PARP inhibitor-induced BER suppression results in unrepaired double-strand breaks and eventual cell death (Abbotts and Wilson, 2017).

Another molecule that can effectively target the DNA repair pathway is DNA-PK, a critical enzyme in the NHEJ pathway, belongs to the PI3K family and plays a critical role in various cellular processes. Selective inhibitors of DNA-PK have demonstrated radio sensitization in preclinical investigations (Shinohara et al., 2005; Davidson et al., 2013; Ismail et al., 2004) Currently, three phase 1 clinical-trial are underway to evaluate the safety and tolerability of a DNA-PK inhibitor (M3814) in combination with palliative RT with or without IT for advanced solid tumors (NCT02516813 and NCT03724890), as well as in combination with curative-intent RT for locally advanced rectal cancer (NCT03770689).

IR-induced DNA damage remains a pivotal component of cancer therapy. Harnessing the molecular vulnerabilities arising from this damage, along with incorporating innovative treatment strategies, is critically important for advancing the field and achieving better outcomes for cancer patients. Advancements in understanding radiation-induced DNA damage and ongoing research into personalized treatment approaches hold promise for improving cancer therapy outcomes. Exploring novel targets and refining treatment modalities will likely contribute to more effective and less toxic cancer treatments in the future.

## Combining DDR with chemotherapy (CT)

Historically, the majority of traditional CT treatments, such as direct agents that damage DNA, have been regarded as immunosuppressive, and one of the most frequent adverse effects of cytotoxic CT that limits dosage is lymphopenia. On the other hand, a growing corpus of experimental and empirical data indicates that certain CT, when administered at recommended dosages, may stimulate immunogenic tumor cell death and influence the tumor microenvironment to support immunity against tumors (Galluzzi et al., 2017). Immunogenic cell death pathways are the first mechanism via which CT has been demonstrated to activate the host immune system. Contrary to apoptosis, which is usually thought to be non-immunogenic, CT may cause cell death and the release of antigens from tumor cells. CT-induced cellular stress increases the immunogenicity of the cell by encouraging the surface expression and secretion of damage-associated molecular patterns (DAMPs) (Klarquist et al., 2014). Apart from cytosolic DNA, which is a potent DAMP, several other cellular proteins have also been observed to function as DAMPs. These include heat-shock proteins, calreticulin, hyaluronan, and high mobility group box 1 (HMGB1). DAMPs that have been released attach to receptors on stromal and cancer cells, triggering a host immune response that is like that of a pathogen. Type I IFN and other chemokines are secreted more readily when DAMP is activated, and DCs need these chemokines to activate tumor-specific CD8^+^ T lymphocytes (Diamond et al., 2011). Additionally, MHC class I and cancer-testis antigens can be expressed more prominently during DNA-damaging CT, while inhibitory mediators such as PD-L1 can be expressed less prominently on the surface of cancer cells (Vereecque et al., 2004). DNA-damaging CTs exert a profound impact on the tumor microenvironment, influencing immune regulatory cell activity and the tumor vasculature, thereby facilitating antitumor immunity. The term “antitumor activity” describes a treatment’s or intervention’s multidimensional capacity to obstruct a tumor cell’s ability to grow, spread, or survive via a variety of methods. This involves the direct destruction of tumor cells through the use of medications used in CT, targeted therapies, and IT, which cause cancer cells to undergo certain cell death processes such as necrosis or apoptosis (Khalaf et al., 2021). Additionally, interventions may hinder tumor cell proliferation and division by disrupting signalling pathways or inhibiting DNA replication, using repair enzymes. Tumor vasculature disruption, aimed at starving tumors of vital nutrients and oxygen by targeting their blood supply, is another strategy (Jing et al., 2019). Furthermore, antitumor immune responses can be activated through ITs, leveraging the body’s defences to identify and eliminate cancer cells or block immune checkpoints hindering antitumor responses. Finally, antitumor strategies may prevent metastasis by impeding the invasion and migration of cancer cells to distant sites. The overarching aim of antitumor approaches is to effectively combat cancer while minimizing harm to healthy tissues, thereby enhancing patient outcomes and quality of life (Said and Ibrahim, 2023). CTs have been demonstrated to downregulate these inhibitory signals in a few contexts, and there are numerous feedback mechanisms that work to limit the host immune response. For example, it has been demonstrated in animal models that medications including gemcitabine, cyclophosphamide, paclitaxel, fludarabine, and 5-fluorouracil decrease Treg or myeloid-derived suppressor cell (MDSC) function (Lutsiak et al., 2005; Zhang et al., 2008). Upregulating DC function is another way to activate the immune system, and CT drugs like cyclophosphamide have been demonstrated to boost DC activity and quantity (Nakahara et al., 2010). These findings underscore the multifaceted effects of DNA-damaging CTs in modulating the tumor microenvironment to promote antitumor immune responses. Perhaps not surprisingly, given the interaction between DNA damaging drugs and the tumor immune response, several lines of research has shown thar cytotoxic CT sensitizes tumors to ICB. For instance, in a mouse model of lung cancer, pre-treatment of the animals with oxaliplatin and cyclophosphamide was sufficient to promote sensitivity to host T cell immunity (Wang et al., 2015). In ovarian cancer, decitabine improved lymphocyte activity and worked in concert with CTLA-4 inhibition.

## Advancement in synergy between DDR and IT

**FIGURE 2:**
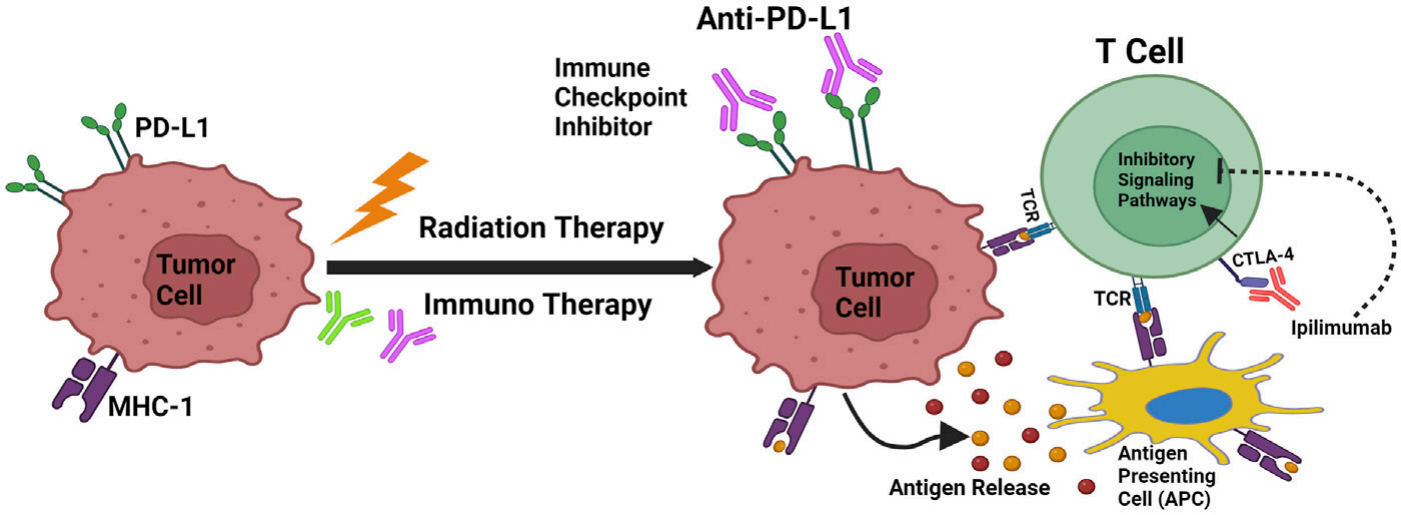
Cancer immunoediting and RT/IT Synergism. Mechanisms that support radiation and immunotherapy’s synergistic effects. Through antigen release, calreticulin activation, and CD47 downregulation, radiation increases the capacity of antigen-presenting cells to transmit tumor antigens to naive T cells. T-Cell Receptors (TCR) engage when MHC-1 is expressed, which leads to the antigen being presented. As PD-L1 and CTLA-4 are upregulated by radiation, immunotherapy that specifically targets these pathways can increase the effectiveness of radiation therapy.

**FIGURE 3:**
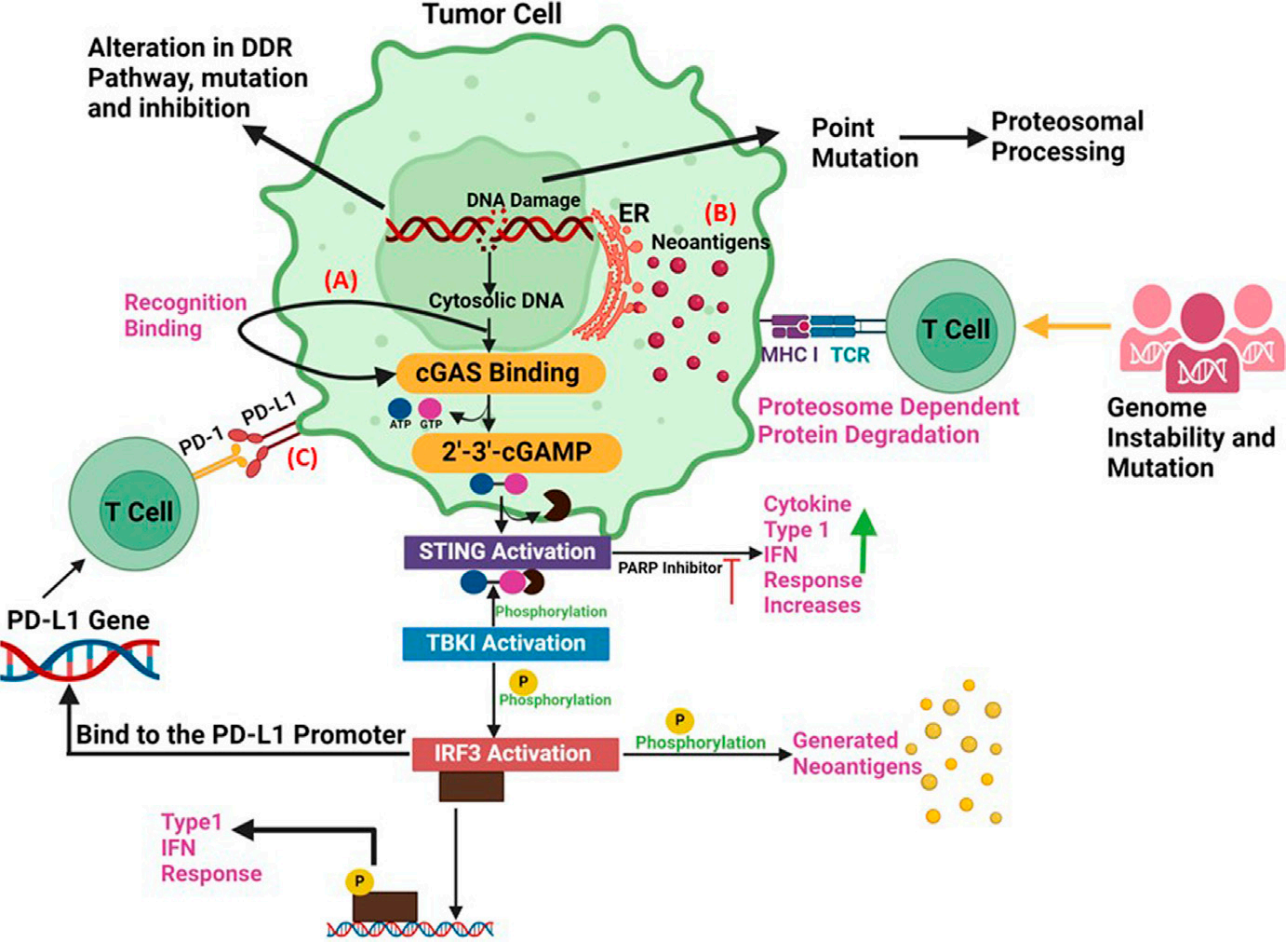
Synergy between DDR and Immunotherapy **(A)** The accumulation of cytoplasmic DNA and activation of the cGAS/STING pathway: DNA damage response defects can increase the cytosolic DNA., that can generate the xGAMP by activation of cGAS. By catalysing the synthesis of cGAMP, which functions as a second messenger in the activation of STING pathway, the cytosolic DNA sensor cGAS can trigger innate immune responses. When the STING pathway is activated, STING undergoes a conformational shift that causes an endoplasmic reticulum to perinuclear endosome shuttle. TBK1 could phosphorylate both IRF3 and STING, which increases the synthesis of type I IFNs. **(B)** Neoantigen Production: Alterations, mutations and inhibition in DDR pathway can promote the production of tumor neoantigens. Deficits in DDR produce neoantigens that enhance tumour identification. According to the neoantigen hypothesis, a non-synonymous mutation modifies an amino acid, resulting in the production of a new peptide. Therefore, the immune system can identify cancer cells lacking DDR as alien. **(C)** PD-L1 upregulation via DNA damage signals: The upregulation of PD-L1 expression is regulated by DNA damage signalling and DDR deficits. Immune checkpoint inhibitors are susceptible to cancer cells treated with PARP inhibitors. ICIs combined with PARP inhibitors show promising results against cancer since PARP inhibitors upregulate the expression of PD-L1 on cancer cells, promote genomic instability, and activate immunological pathways.

Cancer IT represents a promising new therapeutic paradigm to harness the patients’ immune system to eliminate the cancer cells (Kalisz et al., 2019). Both immune evasion and genome instability are characteristic features of cancer. When a proto-oncogene or tumor suppressor gene experiences DNA damage and there are insufficient or no DNA repair mechanisms available to fix the damage, tumorigenesis is often triggered. Immuno evasion subsequently stops the host immune system from identifying these transformed cells. Cancer has proven to respond well to treatments that target immune evasion and genetic instability (Lee et al., 2022). Variations in DNA damage response genes and the resultant genomic instability have a significant role in determining the antigenicity of tumors via both neoantigen-dependent and independent processes (Li and Chen, 2018). As a result, there has been a growing focus on utilizing DDR mutational status as a predictive biomarker for the response to immune checkpoint blockade. This approach aims to enhance patient selection and guide therapeutic choices (Aiello et al., 2020). Similar to RT, DDR deficiency results in heightened DNA damage and an increased tumor mutational burden (TMB) due to the accumulation of point mutations and indels, a hallmark of cancer (Said and Ibrahim, 2023). The higher number of DDR mutations influences the efficacy of IT. Immune checkpoint blockade is a form of IT that prevents the activation of inhibitory immune checkpoints, enabling the immune response to target cancer cells (Dunn et al., 2004). The link between the host immune system and the tumor has been understood for several years, and the ITs aimed at inducing the host immune system to eliminate the tumor cells have demonstrated some degree of clinical effectiveness. For example, therapies include the use of systemic IL2 in metastatic melanoma, renal cell carcinoma, as well as intravesicular *Bacillus* Calmette-Guerin (BCG) in bladder cancer (Klapper et al., 2008). Nonetheless, the use of antibodies against inhibitory signalling molecules on tumour and immune cells in clinical trials has revolutionised the area of cancer IR in the last 5 years. Genomic instability carried on by DDR failures, can activate signalling pathways such as cyclic GMP-AMP synthase-stimulator of interferon genes (cGAS-STING), upregulate the expression of programmed death ligand 1 (PD-L1), and enhance the production of DNA-based neoantigens. In a small number of patients, ICIs such as anti-cytotoxic T-lymphocyte-associated protein 4 (anti-CTLA4) and anti-PD1/PD-L1 antibodies have significantly improved treatment outcomes. Ipilimumab (anti-CTLA-4), an immune checkpoint inhibitor, was the first treatment to show a benefit in survival for patients with metastatic melanoma (Mellman et al., 2011) (shown in Figure 2). Subsequently, there has been proof that therapies targeting the PD-1 pathway, such as PD-1/PD-L1 inhibitors, have shown strong therapeutic efficacy. PD-1 is an immune checkpoint expressed on T cells, while PD-L1 is found on tumor cells (Vereecque et al., 2004; Lutsiak et al., 2005; Zhang et al., 2008). By blocking PD-1/PD-L1 interaction, these therapies unleash the immune system to recognize and attack cancer cells, leading to significant improvements in patient outcomes across various cancer types (Borst et al., 2021). PD-L1 is a binding partner is expressed on antigen presenting cells such as tumour cells and DCs. T cell activation and proliferation are reduced when PD-1 binds to PD-L1 (Chen and Han, 2015; Freeman et al., 2000). PD-1/PD-L1 inhibitors have been extensively evaluated in clinical settings on a variety of cancer types, including colon, lung, and melanoma. The FDA has approved PD-1/PD-L1 inhibitors such as pembrolizumab, nivolumab, durvalumab, and atezolizumab for the treatment of cancer. To date, research has demonstrated that the PD-L1 expression in tumors is controlled by DNA repair and signalling via numerous routes. Endogenous DNA damage in tumors may be continuously produced by oxidative stress or aberrant cell cycling prior to cancer treatment (Katerji and Duerksen-Hughes, 2021). One such pathway involves the activation of interferon regulatory factors (IRFs) and nuclear factor kappa B (NF-κB) in response to DNA damage. These transcription factors can induce the expression of pro-inflammatory cytokines and chemokines, including interferons and interleukins, which promote an inflammatory microenvironment within the tumor. This inflammatory milieu can, in turn, upregulate the expression of PD-L1 on tumor cells, facilitating immune evasion. Moreover, DNA damage-induced signalling pathways, such as the Ataxia-telangiectasia mutated (ATM) and ataxia telangiectasia and Rad3-related (ATR) pathways, can directly regulate PD-L1 expression through various mechanisms. For instance, activation of ATM and ATR kinases can lead to the stabilization of PD-L1 mRNA or the phosphorylation of transcription factors involved in PD-L1 transcriptional regulation (Kakoti et al., 2020). In such situations, immunological signalling may be upregulated by DNA damage responses. However, the immune activity under the circumstances without extra exogenous DNA damage, e.g., prior to RT/CT, is not totally able to overcome malignancies (Zhang et al., 2020). This condition will be expected in case of low TMB/MSI tumors. In contrast, recent studies have demonstrated that several immunological responses, including the release of interferons (IFNs, immune positive response) and PD-L1 upregulation (immune negative response) are generated after DNA damage-associated cancer treatments, such as RT and CT. DNA fragments accumulate in the cytoplasm of cells as a result of DNA damage, and cyclic GMP-AMP synthase (cGAS) is able to identify these fragments. cGAS binds to dsDNA sequences and begins signalling downstream through the STING pathway (Ye et al., 2021), which is a protein that affects immune system modulation. Type I interferon (IFN-1) and other inflammatory cytokines are expressed more when STING stimulates gene transcription via interferon regulatory factor 3 (IRF3) (Figure 3). Furthermore, STING can trigger a transcriptional response via the nuclear factor kappa-light-chain enhancer of activated B cells, including canonical and noncanonical pathways (Motwani et al., 2019). This leads to an increase in DCs presentation of tumor antigen, which in turn intensifies the CD8^+^ T-cell responses.

There are several molecular mechanisms that result in the synergy between IT and RT, improving their combined therapeutic efficacy. By causing immunogenic cell death, RT releases tumor associated antigens, which in turn stimulate T cells and DCs responses. Additionally, it alters the tumour microenvironment by boosting immune cell infiltration and lowering immunosuppressive factors (Yu S. et al., 2022). Type I interferon production is increased upon activation of the cGAS-STING pathway by RT-induced cytosolic DNA, hence augmenting anti-tumor immunity. Additionally, by enhancing DNA damage and boosting the generation of ROS, as well as acting as immunomodulator delivery vehicles, nanoparticles like gold and hafnium oxide augment the effects of RT (Yu R. et al., 2022). Since ICIs like anti-CTLA-4 and anti-PD-1 prevent T cell depletion and maintain their anti-tumor effectiveness, the combination of RT and these agents has demonstrated great potential. These synergistic pathways demonstrate how RT and IT can work synergistically to enhance the immune system’s ability to fight cancer and improve treatment outcomes (Shiravand et al., 2022).

IT combined with DDR-targeted drugs can overcome immune suppression imposed on by DDR abnormalities in cancer cells, thereby establishing antitumor immune responses and enhancing therapeutic results. This synergistic approach has considerable potential to improve overall survival rates and long-term disease control in cancer patients by increasing the patient group eligible for treatment and preventing or delaying the formation of resistance to IT (Wang et al., 2022).

## RadScopal effect

Researchers are exploring a relatively newer concept of ‘RadScopal effect’ that is described as the immunomodulatory effect that uses both high-dose radiations for immune priming in the primary tumor alongside low dose radiation targeted at the secondary tumor to support immune cell infiltration and promote effective tumor eradication. James Welsh proposed this radiation approach by combining high dose RT (HDRT) with low dose RT (LDRT) (Barsoumian et al., 2020a)**.** Low dose radiation may be an effective tool to overcome the limitations of immunosuppressive factors produced by conventional RT. The fundamental mechanisms underlying the discernible impact of low-dose RT might be initiated by the initiation of DNA damage (Mavragani et al., 2015). Studies have shown that low dose RT creates a welcoming environment for the immune cells and promotes an anti-tumor response. Klug and co-workers showed that LDRT shifts pro-tumor M2-macrophages towards the anti-tumor M1-phenotype, increases the infiltration of CD4^+^ T cells and Natural Killer cells, and downregulates the expression of the inhibitory cytokine TGF-β (Klug et al., 2013). A proteomic analysis shows the role of LDRT in the upregulation of stimulatory factors and tumor microenvironment specific cytokines. MIP1α, CD137 (4-1BB), and Granzyme B were upregulated in tumor-infiltrating CD4^+^ T cells, indicating their activation and effector function. Also, in murine lung cancer models, LDRT further enhanced the effectiveness of CPIs such as anti-PD1 and CTLA-4, as confirmed by diminished tumor growth rates and prolonged survival (Patel et al., 2021). A recent clinical study was done in which it was found that, in patients with immune-resistant solid tumors, low-dose RT + high-dose RT safely enhanced lesion-specific responses by enhancing the infiltration of immune cells into the tumor microenvironment**.** Another study done by Herrera et al. endorsed the reasoning behind integrating LDRT with IT for metastatic ovarian cancer (Klug et al., 2013).

Although the radscopal effect, in which targeted radiation therapy incites a systemic immune response against tumors, has great potential for treating cancer, its practical implementation is restricted by various issues. This is a quite uncommon and erratic phenomenon that varies in its occurrence depending on the type of tumor and the environment. Tumors frequently develop immunosuppressive mechanisms that can reduce the efficiency of the radscopal effect, and the best radiation dosages and fractionation schedules to produce it are still unknown. The clinical usefulness of RT is further complicated by difficulties in tracking and evaluating its systemic effects (Wang et al., 2023).

## Clinical benefits of combining RT with IT

The clinical benefits of combining ITs with RT has been demonstrated by many pre-clinical studies. The conventional benefits of adding CT with RT also hold ground for IT addition. RT and IT have been shown to have synergistic benefits by virtue of, the supra-additive effect of the two modalities. To achieve a certain biological effect, lower individual doses are required. Radio-sensitizing properties of IT agents will act on the same principles (Weichselbaum et al., 2017) as well as spatial cooperation. The combination of radio and IT can help overcome the treatment resistance. Tumor stroma and associated macrophages cause T-cell anergy and inefficient T-cells migration into the tumor. It is considered as one of the major reasons behind the resistance to IT (Turgeon et al., 2019). Targeted activation of the tumor microenvironment by combining with RT may overcome this resistance and augment the clinical outcome (Sharma et al., 2017; Waldman et al., 2020). A combination of RT and IT may prevent disease recurrence, as combining them may induce protective “immunologic memory,” which in turn could prevent disease recurrence.

As of now, our understanding of the intricate relationship between radiation and the immune system remains incomplete, yet numerous intriguing observations have emerged. The cytotoxic impact of RT on tumor cells facilitates the generation of tumor neoantigens for T lymphocytes and triggers the release of pro-inflammatory cytokines, thereby fostering an immune response. In preclinical and clinical settings, various groups used the advantageous immunomodulatory properties of radiation to initiate a more potent systemic anticancer immune response against tumors throughout the body (Morris et al., 2016)**.** This treatment approach, *in situ* vaccination, where the patient’s tumor serves as a source of tumor-specific antigens, prompting and broadening a robust antitumor T cell response. Many clinical findings justify combining radiation with immune checkpoint blockade (Deng et al., 2014; Dovedi et al., 2017)^.^ Different studies highlight the use of IR/IT in different types of cancer including melanoma, NSCLC, rectal cancer, etc., as shown in Table 1 (Chen D. et al., 2020). When compared to monotherapy, IRT offers promising avenues for enhancing localized lesion control and inducing the abscopal effect. Here, we review the recent IRT clinical trials and delve into their potential significance in clinical use. Initially, Golden and his team demonstrated that the combination of local RT and granulocyte-macrophage colony-stimulating factor (GM-CSF) indeed yields a substantial objective abscopal effect in individuals afflicted with metastatic solid tumors (Golden et al., 2015).

**TABLE 1:** Pre-clinical and clinical trials using various immunotherapy and different radiation schedule.

Trial	Pre-clinical/Clinical disease	Dose of immune modulatory agent	Dose of RT	Outcome
Barsoumian et al. (2020b)	344SQ lung adenocarcinoma tumors in 129Sv/Ev mice	Intraperitoneally at doses of 50 µg/injection for anti-CTLA-4 and 200 µg/injection for anti-PD1	Primary tumor irradiated locally with 12 Gy/ 3 fractions and secondary tumors were irradiated with low dose RT (two fractions of 1 Gy each) 3 days later.	If tumor burden is high, high-dose RT helps to ‘prime’ T cells at primary tumor site, low-dose RT ‘modulates the stroma’ at secondary metastatic site. Low-dose RT can improve the outcomes of ICI by promoting M1 macrophage polarization, enhancing NK cell infiltration, and reducing TGF-β.
Tang et al. (2017)	Five-arm trial of SBRT with either concurrent or sequential CTLA4 blockade with ipilimumab for patients with metastatic nonmelanoma cancers	Ipilimumab at a dose of 3 mg/kg given every 21 days for a total of 4 doses, concurrent or sequential	SABR to 50 Gy/ 4 fractions	sequential treatment in lung group had highest rate of clinical benefit and no differences in treatment-related adverse events
Kwon et al. (2014)	Metastatic castration-resistant prostate cancer that progressed after docetaxel	Ipilimumab 10 mg/kg administered every 3 weeks for up to four cycles	8 Gy single fraction bone directed RT	reductions in PSA concentration and improved progression-free survival in favour of ipilimumab plus RT
Kiess et al. (2015)	Melanoma patients with brain metastasis	Ipilimumab at a dose of 3 mg/kg or 10 mg/kg given every 21 days for a median of 4 doses, concurrent or sequential (before or after RT)	single fraction SRS for brain metastasis with median dose 21 Gy (15-24 Gy)	OS was significantly better in patients treated with SRS during or before ICI
Sundahl et al. (2019)	Metastatic urothelial carcinoma	Pembrolizumab (200 mg, 3-weekly administered either sequentially or concomitantly	SBRT 3 × 8 Gy, to metastatic lesion	Concurrent administration showed nearly 50% response, while sequential administration of showed nil response
Patel et al. (2021)	Metastatic disease that progressed on IT	Anti PD1, Anti PDL1, CTLA4 inhibitor are used	SBRT (50 Gy /4 fractions or 60–70 Gy/ 10 fractions; 20– 30 Gy/ 5 fractions or 30–45 Gy/ 10 fractions via conventional schedule; Low dose RT consisted of 1–10 Gy total doses delivered in fractions of 0.5–2 Gy	High dose RT ± low dose RT improved lesion-specific response in immune resistant solid tumors with tolerable toxicity.
Peters et al. (2017), Peters et al. (2021)	Single-arm phase II trial in stage III NSCLC	Platinum-based chemotherapy and concurrent RT, along with nivolumab (360 mg, 3-weekly). Nivolumab was continued as consolidation therapy for a maximum of 1 year	66 Gy/ 33 fractions thoracic RT	PFS and OS are higher than the similar cohort of patients
Qin et al. (2020)	Advanced NSCLC	1.2 gm atezolizumab, every 3 weeks	24Gy/ 3 fractions or 30 Gy/ 5 fractions concurrent RT	Overall response rate of 25% and disease control rate of 50%. Incidence of grade 3 A/E similar to that of atezolizumab alone

Abbreviation: RT, Radiotherapy; PD-L1, Programmed death-ligand 1; PD-1, Programmed cell death protein 1; CTLA-4, Cytotoxic T-lymphocyte–associated antigen 4; SBRT, Stereotactic body radiation therapy; SRS, stereotactic radiosurgery; NSCLC, Non-small cell lung cancer; PFS, Progression-free survival; PSA, Prostate specific antigen; OS, Overall survival; A/E, Adverse effects; ICI, Immune checkpoint inhibitor; NK cells, Natural Killer cells; TGF-β, Transforming growth factor beta.

A Phase 1 study was conducted to evaluate the immunologic response and safety induced by autologous DCs in hepatoma patients who received a single fraction of RT. Out of 14 patients, two patients had achieved a partial response (Chi et al., 2005). Shaverdian and co-workers performed a secondary examination of the KEYNOTE-001 trial, revealing that individuals who underwent a combination of RT and pembrolizumab had longer progression-free survival and improved overall survival compared to those who had not undergone prior RT while maintaining a reasonable safety profile (Shaverdian et al., 2017). A prospective trial has been conducted to show that the combination of RT with immune checkpoint blockers has a significant role in the survival of patients (shown in Table 2). Kwon et al. conducted a multicentre phase 3 clinical trial with men who had at least one bone metastasis from castration-resistant prostate cancer that had advanced following docetaxel treatment, based on good preclinical findings in a spontaneous mouse model of prostate cancer. Patients were given either ipilimumab or a placebo after radiation therapy (8 Gy in one portion) for bone metastasis. Comparing ipilimumab to placebo did not affect overall survival (*p* = .053) (Kwon et al., 2014). Patients with good prognostic indicators (no visceral metastases, no anaemia, normal alkaline phosphatase) and treated with ipilimumab exhibited a statistically significant enhancement in survival relative to those receiving the placebo (Kwon et al., 2014). A study conducted by Susan M Domchek et al., focused on patients with germline BRCA1-or BRCA2-mutated metastatic breast cancer, an open-label, multicenter, phase 1/2 basket study was designed to evaluate the safety and efficacy of olaparib in combination with the PD-L1 inhibitor durvalumab. The multicentre, open-label, phase 1/2 MEDIOLA pilot project is evaluating durvalumab with Olaparib in solid tumours. The four groups that were enrolled were germline BRCA-mutated metastatic ovarian cancer, relapsed small-cell lung cancer, germline BRCA-mutated metastatic breast cancer, and metastatic gastric cancer. The cohort with breast cancer is the subject of this summary. Individuals with progressive, HER2-negative metastatic breast cancer who were at least 18 years old (or 19 years old in South Korea) and had germline BRCA1 or BRCA2 mutations were included. After receiving 300 mg of Olaparib twice day for 4 weeks, participants were given 300 mg of Olaparib plus 1.5 g of durvalumab every 4 weeks until the disease progressed. Safety, tolerability, and the 12-week disease control rate were the main outcomes. The study is still underway even though recruitment is over (ClinicalTrials.gov, NCT02734004) (Domchek et al., 2020). In another study, ICIs have shown good results in combination with PARP inhibitors in ovarian cancer. Immunogenomic profiling and single-cell imaging were used on tumor samples from patients enrolled in the phase I/II trial (NCT02657889) integrating pembrolizumab and niraparib. In this study, single-cell spatial analysis demonstrated a significant connection between PD-L1+ macrophages, PD-L1+ tumor cells, and fatigued CD8^+^ T-cells. Additionally, geographical analysis of the two extreme responders revealed distinct clustering of exhausted CD8^+^ T-cells with PD-L1+ macrophages in the first extreme responder, and exhausted CD8^+^ T-cells with cancer cells harbouring genomic PD-L1 and PD-L2 amplification in the second extreme responder (Färkkilä et al., 2020). Nonetheless, the optimal synergistic effects between immune ICIs and RT, encompassing factors like sequence, targeted irradiation sites, dosage, and fractionation, necessitate further exploration and refinement.

**TABLE 2:** Therapies and clinical indications of various USFDA approved immunotherapy agents.

Cancer Type	Name of immunomodulators	Target antibody	Approved indications
Bladder Cancer	Atezolizumab (Peters et al., 2021)	Anti PD-L1 antibody	Advanced urothelial carcinoma
	Avelumab (Grivas et al., 2023)	Anti PD-L1 antibody	Advanced bladder cancer, including as first-line maintenance therapy after chemotherapy
	Dostarlimab (Kasherman et al., 2021)	Anti PD-1 antibody	Advanced bladder cancer that has dMMR
	Nivolumab (Rhea and Aragon-Ching et al., 2021)	Anti PD-1 antibody	Advanced bladder cancer
	Pembrolizumab (Crist et al., 2019)	Anti PD-1 antibody	Advanced bladder cancer
Lung cancer	Atezolizumab (Farid and Liu, 2020)	Anti PD-L1 antibody	NSCLC and SCLC, including as a first-line therapy in combination with chemotherapy.
	Cemiplimab (Ahn and Nagasaka, 2023)	Anti PD-1 antibody	Advanced NSCLC
	Dostarlimab (Lim et al., 2023)	Anti PD-1 antibody	Advanced lung cancer that has dMMR
	Durvalumab (Saad et al., 2022)	blocks the interaction of PD-L1 with PD-1	Stage III NSCLC who have completed chemoradiation, as well as patients with advanced SCLC in combination with chemotherapy.
	Ipilimumab (Ye et al., 2022)	Anti CTLA-4 antibody	Approved, in combination with nivolumab, as a first-line treatment for patients with advanced NSCLC and mesothelioma
	Nivolumab (Ye et al., 2022)	Anti PD-1 antibody	Advanced NSCLC and mesothelioma in combination with ipilimumab, with or without chemotherapy
	Pembrolizumab (Saad et al., 2022)	Anti PD-1 antibody	Advanced NSCLC, including as a first-line therapy its own or in combination with chemotherapy
Brain Tumor, Breast, Uterine, cervical, pancreatic, colorectal and Gastric cancer, Soft tissue sarcoma	Dostarlimab (Andre et al., 2023)	Anti PD-1 antibody	Advanced brain/nervous system cancer that exhibit dMMR
	Pembrolizumab (Yan et al., 2024)	Anti PD-1 antibody	Advanced brain or nervous system cancers that have high MSI-H, dMMR, or high TMB-H

Abbreviation: USFDA, United States Food and Drug Administration; PD-L1, Programmed death-ligand 1; PD-1, Programmed cell death protein 1; dMMR, Deficient mismatch repair; NSCLC, Non-small cell lung cancer; SCLC, Small cell lung carcinoma; MSI-H, microsatellite instability-high; TMB-H- tumor mutational burden-high.

## Optimal RT dose-fractionation schedule

The immune response induced by RT is “dose-dependent” and the optimum dose fractionation schedule is debatable. Several pre-clinical studies suggest higher doses per fraction can achieve better outcomes, maintaining low Treg numbers and a greater number of host immune cells infiltrating the tumors (Chen Y. et al., 2020). A series of clinical studies and reports also suggested that a combination of IT with stereotactic body radiation therapy (SBRT) produces excellent clinical results with tolerable toxicity (Chen Y. et al., 2020; Menon et al., 2019). In 2012, the first such case report demonstrated that ipilimumab in conjunction with SBRT (28.5 Gy/three fractions) produces a good local response at paraspinal metastatic mass but also regression of distant lesions away from the radiation field in a patient with metastatic melanoma (Tang et al., 2017)

A pooled analysis of two trials (PEMBRO-RT and MDACC) demonstrated higher doses of radiation along with pembrolizumab can significantly increase responses in patients with metastatic NSCLC, as shown in Table 2 (Reisz et al., 2014; Huang et al., 2013). In the PEMBRO-RT trial, pembrolizumab was given sequentially less than 1 week after the last dose of radiotherapy (24 Gy/three fractions), whereas in the MDACC trial, pembrolizumab was given concurrently with the radiotherapy (50 Gy/four fractions or 45 Gy/15 fractions). These results also corroborate the other similar clinical trials confirming the safety and efficacy of SBRT along with ITs. Nevertheless, few studies involving conventional doses of RT with IT showed no significant difference in outcome and hence can be effectively administered when SBRT is not feasible (Ferris et al., 2022; Elbers et al., 2020). The heterogeneity of tumor cells and their radiosensitivity probably contribute to differential responses to dose fractionation schedules of RT.

Selecting the optimal dose of RT involves considering factors such as tumor characteristics, treatment intent (curative vs palliative), radiation sensitivity, normal tissue tolerance, treatment schedule, patient factors, and clinical guidelines. Treatment planning tools and sophisticated imaging methods aid in ensuring that the tumor receives a precise dose while neighbouring healthy tissues are spared (Wang et al., 2023). Close monitoring during treatment enables adjustments based on toxicity and tumor response. Radiation oncologists optimize tumor control while minimizing side effects by incorporating these factors into the dose of radiation therapy that they administer to each patient.

Tumors may react differently to different RT schedules depending on differences in their radiation sensitivity and features. Radiation may more readily destroy some cells while sparing more resilient ones. Plans for radiation therapy can be adjusted to take these variations into consideration in order to maximize treatment efficacy and reduce harm to healthy tissue (Wang et al., 2023).

## The optimal timing for RT in combination with IT

The selection of optimal timing of RT and the evaluation of the safety and efficacy of IT as “concurrent” or “sequential” warrant critical clinical judgment. Different types of IT target different immunological pathways, and radiation can exert its effect differentially based on the dose and fractionation. Therefore, a single strategy to achieve the greatest synergistic effects is not feasible for multiple cancers. IT drugs are typically administered concurrently or after RT to allow newly recruited T lymphocytes to effectively kill tumor cells both at the main site and at distant regions after being exposed to tumor antigens (Weichselbaum et al., 2017). The PACIFIC study demonstrated that Durvalumab^®^ can be safely used as a maintenance medication with a survival benefit and limited toxicity following chemoradiotherapy (Spigel et al., 2022). However, a subset analysis of the PACIFIC trial suggests that initiating Durvalumab^®^ within 2 weeks after completing chemoradiotherapy appeared to have greater progression free survival (PFS) rather than starting after 2 weeks of chemoradiation (Faivre-Finn et al., 2021). While sequential administration of IT followed by RT have been well established, many studies are enthusiastically investigating the effect of concurrent administration of IT. A phase II trial of concurrent Atezolizumab^®^ with chemoradiation for patients with unresectable NSCLC, showed that concurrent RT with Atezolizumab^®^ followed by the consolidation and maintenance of Atezolizumab^®^ therapy is feasible, and no added toxicities are reported as compared with historical rates (Durm et al., 2020).

## Adverse effects associated with RT/IT

The adverse effects of individual IT agents and RT-induced toxicities may overlap and can act as limiting factors for the combined use of these two modalities. The most severe injury caused by thoracic RT is radiation-induced lung injury, which manifests as radiation pneumonitis and radiation pulmonary fibrosis (Arroyo-Hernández et al., 2021). Usually, radiation pneumonitis occurs in 6 months after RT, and radiation pulmonary fibrosis occurs as a late side effect following RT (Kalisz et al., 2019; Zhai et al., 2020). Radiation-induced heart disease for thoracic RT and liver injury for upper abdominal RT are two other serious complications that are examples of RT-induced normal tissue toxicities. On the other hand, IT, particularly ICIs, can lead to unique toxicities such as checkpoint inhibitor pneumonitis, colitis, hepatitis, endocrinopathies, and dermatologic toxicity due to their mechanisms of action. Clinical trials suggest that IT and RT is generally well tolerated with mild to moderate toxicities (grades 1-2), while severe toxicities (grade 3 or 4) are relatively rare (Kennedy and Salama, 2020; Zhang et al., 2021). Although, PACIFIC trial laid the framework for the sequential use of durvalumab after chemoradiation with an acceptable range of toxicity in NSCLC patients; HOPE-005/CRIMSON study raised a red flag by reporting a large percentage of pneumonitis through a real-world survey (Saito et al., 2021)0). The survey documented overall 83% of any grade of pneumonitis and 34%, 7%, and 1% of the patients developing symptomatic pneumonitis, ≥ grade 3 pneumonitis, and fatal (i.e., grade 5) pneumonitis, respectively, after receiving durvalumab consolidation therapy (Kalisz et al., 2019). A trial by Hoosier Cancer Research Network LUN 14-179 reported 17.2% symptomatic pneumonitis after receiving concurrent chemoradiation followed by consolidation pembrolizumab in patients with stage III NSCLC (Hiniker et al., 2016) Thereby, IT and radiation dysregulate each other’s immunological backgrounds and enhance immune mediated adverse effects, which can affect any organ and lead to fatal complications. There is a need to select an optimum dosing schedule for the combination of RT and IT, for which larger clinical trials are needed.

Quantitative evaluations, including odds ratios (OR), Hazard Ratio or relative risks (RR), are crucial in illustrating the advantages and possible drawbacks of combining RT and IT with other treatment types. To quantify the effects of treatment, a study comparing the safety and effectiveness of radiation alone versus concurrent CT and IT after radiation for patients with locally advanced head and neck cancer may report an OR for overall survival and an HR for progression-free survival (McGovern et al., 2019). A meta-analysis evaluating the risk of pneumonitis in lung cancer patients receiving IT in addition to RT could serve as another example. This study’s analysis of the relative risk (RR) of pneumonitis development between the radiation alone and combination groups offers important information about the safety profile of the combined approach. These quantitative measurements aid in the decision-making process for researchers and doctors when it comes to risk management procedures and treatment plans (Chen et al., 2023).

## Conclusion and future perspective

There is a plethora of evidence, that combination of IT and RT can produce a significantly better outcome, often translating into a survival benefit for many solid malignancies. Traditionally, advanced and/or metastatic lung carcinoma showed a dismal prognosis and contributes to the highest cancer-related mortality globally. Recently, IT has shown promising results in these patients as second or beyond line of management. Many IT agents are approved by FDA, that act by inhibiting PD-1 and PDL-1 receptors and are demonstrated to increase survival and quality of life without considerable toxicity. On the other hand, RT also has a sustainable, definitive role in inoperable, non-metastatic lung cancer. Palliative radiation can also be used to ameliorate symptoms of advanced malignancy, i.e., haemoptysis, local pain, and superior vena cava syndrome. In these contexts, it is obvious that oncologists frequently encounter scenarios, where a combination of RT and IT is to be considered to aim for a better outcome for patients with advanced lung cancer.

Significant advancements have been achieved in comprehending the possible connections between DDR and immunity. In the presence of external or endogenous DNA damage, a defective or downregulated DDR pathway permits genomic instability, and the tumor microenvironment also plays a role in controlling genomic instability via DDR pathways. Alterations in the open reading frames of proteins can lead to the breakdown of aberrant proteins, which in turn can produce neoantigens through modifications of the DDR pathways. These mutations may be caused by an MMR deficit, poor DNA replication, or endogenous oxidative DNA damage. DSBs trigger DDR signalling in response to CT and/or RT. Tumor cells that have activated ATM-ATR/CHK1 may have an overexpression of PD-L1. This pathway, which is involved in the response to DNA damage, can activate immunological checkpoint molecules such as PD-L1, which aids in the immune system’s evasion of tumor cells. Therefore, targeting this pathway alongside with IT may be beneficial in certain cases to enhance the effectiveness of cancer treatment. Moreover, DNA fragments that trigger the STING pathway can also be produced by cells that have DSBs, which are detectable by cGAS. Cancer cells may perish as a result of excessive DNA damage, which would release DAMP and trigger an immunological response. Consequently, there is great potential for using DDR pathways and their modifications in cancer IT.

The addition of RT to IT enhances the clinical effectiveness, for example,: Tyrosine kinase inhibitor (TKI), namely, gefitinib, exhibits a radiosensitizing effect, and combination with radiation showed modest improvement in advanced epidermal growth factor receptor (EGFR) mutant lung cancers in many clinical trials. It is expected that a similar strategy with IT drugs will also produce significant benefits for the selected patients. Secondary analysis of the KEYNOTE-001 trial has confirmed that patients with advanced NSCLC previously treated with RT yielded longer PFS and overall survival (OS) with pembrolizumab as compared to patients who did not receive prior RT, with a clinically acceptable safety profile. The strong evidence derived from the PACIFIC trial recommended that, continuation of the PD-L1 antibody, namely, durvalumab, can significantly increase the survival of patients with locally advanced, unresectable NSCLC after the completion of chemoradiotherapy. Hence, there might be substantial debate about the sequence, dose of the radiation, fractionation schedule, and optimal choice of IT, but the fact that concurrent or sequential use of RT enhances antitumor immune responses is well established.

Therefore, many pre-clinical and clinical trials have confirmed the safety and efficacy of the combined use of IT and RT that act upon the principle of synergism. An optimal choice of dose, fraction, and IT agents can have immense potential to widen the horizons of cancer treatment in the near future. Local radiation certainly possesses systemic immunomodulatory effects and judicious combined use of IT can augment the effect, and overcome challenges such as disease recurrence and resistance to treatment. However, more mature data from randomised clinical trials is required to firmly establish this novel strategy of cancer treatment.

In recent years, patient care has significantly improved due to the recent developments in our knowledge of IT and the DDR. One of the most reliable indicators of the response to ICI appears to be a defect in the DNA repair mechanisms. Furthermore, the information that is now available demonstrates that RT-induced DNA damage is a major factor in inducing an immune response that regulates the growth of tumours. Gaining more insight into the relationship between the DDR and tumor immunity will help us combine IT and RT more effectively. It will also make it easier to introduce new treatments, such as direct DDR targeting, to enhance the prognosis of cancer patients.

By personalizing treatments for each patient based on biomarkers and genetic profiling to forecast responses to combination RT and IT, personalized medicine in oncology has completely transformed the way cancer is being treated. To stratify patients for ICIs such as pembrolizumab and nivolumab, biomarkers such as PD-L1 expression levels are essential. Furthermore, genomic screening finds relevant mutations, like BRAF in melanoma, that impact treatment outcomes and direct therapeutic choices. Tumor microenvironment (TME) profiling sheds light on immunogenicity and the response to IT, including immune cell infiltration and the cytokine milieu. Integrating these data not only refines patient selection but also aids in treatment planning and monitoring, optimizing outcomes in combined RT and IT strategies. Ongoing research efforts aim to identify novel biomarkers and elucidate their roles, further advancing the precision and efficacy of personalized oncology approaches. As we delve deeper into understanding the intricate interplay between the tumor, immune system, and radiation response, personalized medicine continues to evolve, offering hope for improved outcomes and better quality of life for cancer patients. To predict the results of RT and IT, a variety of models and tools are used in pre-clinical and clinical research. These include pharmacodynamic models, systems biology models, and machine learning techniques. Pharmacodynamic models facilitate understanding how drug concentration and biological effect relate to one another, which makes it easier to comprehend how the body behaves towards RT and IT. Systems biology models examine the dynamics of tumor microenvironments and molecular pathways by integrating multi-scale biological data, offering insights into therapeutic results. Large-scale datasets are analysed by machine learning algorithms to find patterns and forecast individualized treatment responses. This allows for the optimization of treatment regimens and the discovery of biomarkers linked to treatment resistance or efficacy. When combined, these computational methods improve our knowledge of the interactions between RT and IT, enabling the development of individualized treatment plans and leading to better patient results. Monitoring techniques that are comprehensive and systematic can be used to undertake long-term follow-up with patients participating in clinical studies. This entails scheduling frequent follow-up visits with patients in order to evaluate the results of their treatments and their general health over time. In order to monitor therapy responses, identify any problems or late effects, and measure long-term survival, patient data, including clinical assessments, imaging investigations, laboratory tests, and patient-reported outcomes, are gathered at predetermined intervals.

Reporting criteria and standardised assessment tools are frequently used to guarantee accurate and reliable data collection. This makes it possible for researchers to compare results in an efficient manner and helps to ensure uniformity in data collection across many clinical trial sites. Furthermore, data management systems and electronic health records are used to effectively store and interpret longitudinal patient data. Furthermore, for long-term follow-up in clinical studies to be successful, patient participation and engagement are crucial.

## References

[B1] Abbotts R. Wilson D. M. (2017). Coordination of DNA single strand break repair. Free Radic. Biol. Med. 107, 228–244. 10.1016/j.freeradbiomed.2016.11.039 27890643 PMC5443707

[B2] Ahn J. Nagasaka M. (2023). Spotlight on cemiplimab-rwlc in the treatment of non-small cell lung cancer (NSCLC): focus on patient selection and considerations. Cancer Manag. Res. 15, 627–634. PMID: 37457376; PMCID: PMC10349595. 10.2147/CMAR.S325856 37457376 PMC10349595

[B3] Aiello M. M. Solinas C. Santoni M. Battelli N. Restuccia N. Latteri F. (2020). Excision repair cross complementation group 1 single nucleotide polymorphisms and nivolumab in advanced non-small cell lung cancer. Front. Oncol. 10, 1167. 10.3389/fonc.2020.01167 32983959 PMC7493643

[B4] André T. Berton D. Curigliano G. Sabatier R. Tinker A. V. Oaknin A. (2023). Antitumor activity and safety of dostarlimab monotherapy in patients with mismatch repair deficient solid tumors: a nonrandomized controlled trial. JAMA Netw. Open 6 (11), e2341165. PMID: 37917058; PMCID: PMC10623195. 10.1001/jamanetworkopen.2023.41165 37917058 PMC10623195

[B5] Arroyo-Hernández M. Maldonado F. Lozano-Ruiz F. Muñoz-Montaño W. Nuñez-Baez M. Arrieta O. (2021). Radiation-induced lung injury: current evidence. BMC Pulm. Med. 21 (1), 9. 10.1186/s12890-020-01376-4 33407290 PMC7788688

[B6] Barakat K. H. Gajewski M. M. Tuszynski J. A. (2012). DNA polymerase beta (pol β) inhibitors: a comprehensive overview. Drug Discov. Today 17 (15–16), 913–920. 10.1016/j.drudis.2012.04.008 22561893

[B7] Barsoumian H. B. Ramapriyan R. Younes A. I. Caetano M. S. Menon H. Comeaux N. I. (2020a). Low-dose radiation treatment enhances systemic antitumor immune responses by overcoming the inhibitory stroma. J. Immunother. Cancer. 8 (2), e000537. 10.1136/jitc-2020-000537 33106386 PMC7592253

[B8] Barsoumian H. B. Ramapriyan R. Younes A. I. Caetano M. S. Menon H. Comeaux N. I. (2020b). Low-dose radiation treatment enhances systemic antitumor immune responses by overcoming the inhibitory stroma. J. Immunother. Cancer 8 (2), e000537. 10.1136/jitc-2020-000537 33106386 PMC7592253

[B9] Borst J. Busselaar J. Bosma D. M. T. Ossendorp F. (2021). Mechanism of action of PD-1 receptor/ligand targeted cancer immunotherapy. Eur. J. Immunol. 51 (8), 1911–1920. 10.1002/eji.202048994 34106465 PMC8453912

[B10] Burma S. Chen B. P. C. Chen D. J. (2006). Role of non-homologous end joining (NHEJ) in maintaining genomic integrity. DNA Repair (Amst) 5 (9–10), 1042–1048. 10.1016/j.dnarep.2006.05.026 16822724

[B11] Ceccaldi R. Liu J. C. Amunugama R. Hajdu I. Primack B. Petalcorin M. I. R. (2015). Homologous-recombination-deficient tumours are dependent on Polθ-mediated repair. Nature 518 (7538), 258–262. 10.1038/nature14184 25642963 PMC4415602

[B12] Chabner B. A. Roberts T. G. (2005). Timeline: chemotherapy and the war on cancer. Nat. Rev. Cancer 5 (1), 65–72. 10.1038/nrc1529 15630416

[B13] Chen D. Menon H. Verma V. Guo C. Ramapriyan R. Barsoumian H. (2020a). Response and outcomes after anti-CTLA4 versus anti-PD1 combined with stereotactic body radiation therapy for metastatic non-small cell lung cancer: retrospective analysis of two single-institution prospective trials. J. Immunother. Cancer 8 (1), e000492. 10.1136/jitc-2019-000492 31996395 PMC7057428

[B14] Chen F. Niu J. Wang M. Zhu H. Guo Z. (2023). Re-evaluating the risk factors for radiation pneumonitis in the era of immunotherapy. J. Transl. Med. 21, 368. 10.1186/s12967-023-04212-5 37287014 PMC10246421

[B15] Chen L. Han X. (2015). Anti-PD-1/PD-L1 therapy of human cancer: past, present, and future. J. Clin. Invest. 125 (9), 3384–3391. 10.1172/JCI80011 26325035 PMC4588282

[B16] Chen Y. Gao M. Huang Z. Yu J. Meng X. (2020b). SBRT combined with PD-1/PD-L1 inhibitors in NSCLC treatment: a focus on the mechanisms, advances, and future challenges. J. Hematol. Oncol. 13 (1), 105. 10.1186/s13045-020-00940-z 32723363 PMC7390199

[B17] Chi K. H. Liu S. J. Li C. P. Kuo H. P. Wang Y. S. Chao Y. (2005). Combination of conformal radiotherapy and intratumoral injection of adoptive dendritic cell immunotherapy in refractory hepatoma. J. Immunother. 28 (2), 129–135. 10.1097/01.cji.0000154248.74383.5e 15725956

[B18] Crist M. Iyer G. Hsu M. Huang W. C. Balar A. V. (2019). Pembrolizumab in the treatment of locally advanced or metastatic urothelial carcinoma: clinical trial evidence and experience. Ther. Adv. Urol. 11, 1756287219839285. PMID: 31057668; PMCID: PMC6452591. 10.1177/1756287219839285 31057668 PMC6452591

[B19] Curtin N. J. (2023). Targeting the DNA damage response for cancer therapy. Biochem. Soc. Trans. 51 (1), 207–221. 10.1042/BST20220681 36606678 PMC9988002

[B20] Dagar G. Kumar R. Yadav K. K. Singh M. Pandita T. K. (2023). Ubiquitination and deubiquitination: implications on cancer therapy. Biochim. Biophys. Acta Gene Regul. Mech. 1866, 194979. 10.1016/j.bbagrm.2023.194979 37633647

[B21] Das C. Adhikari S. Bhattacharya A. Chakraborty S. Mondal P. Yadav S. S. (2023). Epigenetic-metabolic interplay in the DNA damage response and therapeutic resistance of breast cancer. Cancer Res. 83 (5), 657–666. 10.1158/0008-5472.CAN-22-3015 36661847 PMC11285093

[B22] Davalli P. Marverti G. Lauriola A. D’Arca D. (2018). Targeting oxidatively induced DNA damage response in cancer: opportunities for novel cancer therapies. Oxid. Med. Cell Longev. 2018, 2389523. 10.1155/2018/2389523 29770165 PMC5892224

[B23] Davidson D. Amrein L. Panasci L. Aloyz R. (2013). Small molecules, inhibitors of DNA-PK, targeting DNA repair, and beyond. Front. Pharmacol. 4 (5), 5. 10.3389/fphar.2013.00005 23386830 PMC3560216

[B24] Deckbar D. Jeggo P. A. Löbrich M. (2011). Understanding the limitations of radiation-induced cell cycle checkpoints. Crit. Rev. Biochem. Mol. Biol. 46 (4), 271–283. 10.3109/10409238.2011.575764 21524151 PMC3171706

[B25] Deng L. Liang H. Burnette B. Beckett M. Darga T. Weichselbaum R. R. (2014). Irradiation and anti-PD-L1 treatment synergistically promote antitumor immunity in mice. J. Clin. Invest. 124 (2), 687–695. 10.1172/JCI67313 24382348 PMC3904601

[B26] Diamond M. S. Kinder M. Matsushita H. Mashayekhi M. Dunn G. P. Archambault J. M. (2011). Type I interferon is selectively required by dendritic cells for immune rejection of tumors. J. Exp. Med. 208 (10), 1989–2003. 10.1084/jem.20101158 21930769 PMC3182061

[B27] Domchek S. M. Postel-Vinay S. Im S. A. Park Y. H. Delord J. P. Italiano A. (2020). Olaparib and durvalumab in patients with germline BRCA-mutated metastatic breast cancer (MEDIOLA): an open-label, multicentre, phase 1/2, basket study. Lancet Oncol. 21 (9), 1155–1164. 10.1016/S1470-2045(20)30324-7 32771088

[B28] Dovedi S. J. Cheadle E. J. Popple A. L. Poon E. Morrow M. Stewart R. (2017). Fractionated radiation therapy stimulates antitumor immunity mediated by both resident and infiltrating polyclonal T-cell populations when combined with PD-1 blockade. Clin. Cancer Res. 23 (18), 5514–5526. 10.1158/1078-0432.CCR-16-1673 28533222

[B29] Dunn G. P. Old L. J. Schreiber R. D. (2004). The three Es of cancer immunoediting. Annu. Rev. Immunol. 22, 329–360. 10.1146/annurev.immunol.22.012703.104803 15032581

[B30] Durm G. A. Jabbour S. K. Althouse S. K. Liu Z. Sadiq A. A. Zon R. T. (2020). A phase 2 trial of consolidation pembrolizumab following concurrent chemoradiation for patients with unresectable stage III non-small cell lung cancer: Hoosier Cancer Research Network LUN 14-179. Cancer. 126:(19), 4353–4361. 10.1002/cncr.33083 32697352 PMC10865991

[B31] Eisbruch A. Marsh L. H. Martel M. K. Ship J. A. Ten Haken R. Pu A. T. (1998). Comprehensive irradiation of head and neck cancer using conformal multisegmental fields: assessment of target coverage and noninvolved tissue sparing. Int. J. Radiat. Oncol. Biol. Phys. 41 (3), 559–568. 10.1016/s0360-3016(98)00082-0 9635702

[B32] Elbers J. B. W. Al-Mamgani A. Tesseslaar M. E. T. van den Brekel M. W. M. Lange C. A. H. van der Wal J. E. (2020). Immuno-radiotherapy with cetuximab and avelumab for advanced stage head and neck squamous cell carcinoma: results from a phase-I trial. Radiother. Oncol. 142, 79–84. 10.1016/j.radonc.2019.08.007 31563412

[B33] Elshaikh M. Ljungman M. Ten Haken R. Lichter A. S. (2006). Advances in radiation oncology. Annu. Rev. Med. 57, 19–31. 10.1146/annurev.med.57.121304.131431 16409134

[B34] Faivre-Finn C. Spigel D. R. Senan S. Langer C. Perez B. A. Özgüroğlu M. (2021). Impact of prior chemoradiotherapy-related variables on outcomes with durvalumab in unresectable Stage III NSCLC (PACIFIC). Lung Cancer 151, 30–38. 10.1016/j.lungcan.2020.11.024 33285469

[B35] Farid S. Liu S. V. (2020). Chemo-immunotherapy as first-line treatment for small-cell lung cancer. Ther. Adv. Med. Oncol. 12, 1758835920980365. PMID: 33414848; PMCID: PMC7750570. 10.1177/1758835920980365 33414848 PMC7750570

[B36] Färkkilä A. Gulhan D. C. Casado J. Jacobson C. A. Nguyen H. Kochupurakkal B. (2020). Immunogenomic profiling determines responses to combined PARP and PD-1 inhibition in ovarian cancer. Nat. Commun. 11 (1), 1459. 10.1038/s41467-020-15315-8 32193378 PMC7081234

[B37] Ferris R. L. Moskovitz J. Kunning S. Ruffin A. T. Reeder C. Ohr J. (2022). Phase I trial of cetuximab, radiotherapy, and ipilimumab in locally advanced head and neck cancer. Clin. Cancer Res. 28 (7), 1335–1344. 10.1158/1078-0432.CCR-21-0426 35091445 PMC9164766

[B38] Fortini P. Dogliotti E. (2007). Base damage and single-strand break repair: mechanisms and functional significance of short- and long-patch repair subpathways. DNA Repair (Amst). 6 (4), 398–409. 10.1016/j.dnarep.2006.10.008 17129767

[B39] Fousteri M. Mullenders L. H. F. (2008). Transcription-coupled nucleotide excision repair in mammalian cells: molecular mechanisms and biological effects. Cell Res. 18 (1), 73–84. 10.1038/cr.2008.6 18166977

[B40] Freeman G. J. Long A. J. Iwai Y. Bourque K. Chernova T. Nishimura H. (2000). Engagement of the PD-1 immunoinhibitory receptor by a novel B7 family member leads to negative regulation of lymphocyte activation. J. Exp. Med. 192 (7), 1027–1034. 10.1084/jem.192.7.1027 11015443 PMC2193311

[B41] Galluzzi L. Buqué A. Kepp O. Zitvogel L. Kroemer G. (2017). Immunogenic cell death in cancer and infectious disease. Nat. Rev. Immunol. 17 (2), 97–111. 10.1038/nri.2016.107 27748397

[B42] Garland K. M. Sheehy T. L. Wilson J. T. (2022). Chemical and biomolecular strategies for STING pathway activation in cancer immunotherapy. Chem. Rev. 122 (6), 5977–6039. 10.1021/acs.chemrev.1c00750 35107989 PMC8994686

[B43] Giglia-Mari G. Zotter A. Vermeulen W. (2011). DNA damage response. Cold Spring Harb. Perspect. Biol. 3 (1), a000745. 10.1101/cshperspect.a000745 20980439 PMC3003462

[B44] Golden E. B. Chhabra A. Chachoua A. Adams S. Donach M. Fenton-Kerimian M. (2015). Local radiotherapy and granulocyte-macrophage colony-stimulating factor to generate abscopal responses in patients with metastatic solid tumours: a proof-of-principle trial. Lancet Oncol. 16 (7), 795–803. 10.1016/S1470-2045(15)00054-6 26095785

[B45] Grivas P. Grande E. Davis I. D. Moon H. H. Grimm M. O. Gupta S. (2023). Avelumab first-line maintenance treatment for advanced urothelial carcinoma: review of evidence to guide clinical practice. ESMO Open 8 (6), 102050. Epub 2023 Oct 12. PMID: 37976999; PMCID: PMC10685024. 10.1016/j.esmoop.2023.102050 37976999 PMC10685024

[B46] Groelly F. J. Fawkes M. Dagg R. A. Blackford A. N. Tarsounas M. (2023). Targeting DNA damage response pathways in cancer. Nat. Rev. Cancer 23 (2), 78–94. 10.1038/s41568-022-00535-5 36471053

[B47] Guo G. S. Zhang F. M. Gao R. J. Delsite R. Feng Z. H. Powell S. N. (2011). DNA repair and synthetic lethality. Int. J. Oral Sci. 3 (4), 176–179. 10.4248/IJOS11064 22010575 PMC3469974

[B48] Hiniker S. M. Reddy S. A. Maecker H. T. Subrahmanyam P. B. Rosenberg-Hasson Y. Swetter S. M. (2016). A prospective clinical trial combining radiation therapy with systemic immunotherapy in metastatic melanoma. Int. J. Radiat. Oncol. Biol. Phys. 96 (3), 578–588. 10.1016/j.ijrobp.2016.07.005 27681753 PMC5077166

[B49] Hoeijmakers J. H. (1993). Nucleotide excision repair. II: from yeast to mammals. Trends Genet. 9 (6), 211–217. 10.1016/0168-9525(93)90121-w 8337762

[B50] Huang J. Zhou Y. Zhang H. Qu T. Mao Y. Zhu H. (2013). A phase II study of biweekly paclitaxel and cisplatin chemotherapy for recurrent or metastatic esophageal squamous cell carcinoma: ERCC1 expression predicts response to chemotherapy. Med. Oncol. 30 (1), 343. 10.1007/s12032-012-0343-4 23263828

[B51] Ismail I. H. Mårtensson S. Moshinsky D. Rice A. Tang C. Howlett A. (2004). SU11752 inhibits the DNA-dependent protein kinase and DNA double-strand break repair resulting in ionizing radiation sensitization. Oncogene 23 (4), 873–882. 10.1038/sj.onc.1207303 14661061

[B52] Jeggo P. Lavin M. F. (2009). Cellular radiosensitivity: how much better do we understand it? Int. J. Radiat. Biol. 85 (12), 1061–1081. 10.3109/09553000903261263 19995233

[B53] Jing X. Yang F. Shao C. Wei K. Xie M. Shen H. (2019). Role of hypoxia in cancer therapy by regulating the tumor microenvironment. Mol. Cancer 18 (1), 157. 10.1186/s12943-019-1089-9 31711497 PMC6844052

[B54] Jurkovicova D. Neophytou C. M. Gašparović A. Č. Gonçalves A. C. (2022). DNA damage response in cancer therapy and resistance: challenges and opportunities. Int. J. Mol. Sci. 23 (23), 14672. 10.3390/ijms232314672 36499000 PMC9735783

[B55] Kakoti S. Sato H. Laskar S. Yasuhara T. Shibata A. (2020). DNA repair and signaling in immune-related cancer therapy. Front. Mol. Biosci. 7, 205. 10.3389/fmolb.2020.00205 33102516 PMC7506057

[B56] Kalisz K. R. Ramaiya N. H. Laukamp K. R. Gupta A. (2019). Immune checkpoint inhibitor therapy-related pneumonitis: patterns and management. Radiographics 39 (7), 1923–1937. 10.1148/rg.2019190036 31584861

[B57] Kasherman L. Ahrari S. Lheureux S. (2021). Dostarlimab in the treatment of recurrent or primary advanced endometrial cancer. Future Oncol. 17 (8), 877–892. Epub 2020 Nov 30. PMID: 33251877. 10.2217/fon-2020-0655 33251877

[B58] Katerji M. Duerksen-Hughes P. J. (2021). DNA damage in cancer development: special implications in viral oncogenesis. Am. J. Cancer Res. 11 (8), 3956–3979. 34522461 PMC8414375

[B59] Kennedy L. B. Salama A. K. S. (2020). A review of cancer immunotherapy toxicity. CA Cancer J. Clin. 70 (2), 86–104. 10.3322/caac.21596 31944278

[B60] Khalaf K. Hana D. Chou J. T. T. Singh C. Mackiewicz A. Kaczmarek M. (2021). Aspects of the tumor microenvironment involved in immune resistance and drug resistance. Front. Immunol. 12, 656364. 10.3389/fimmu.2021.656364 34122412 PMC8190405

[B61] Kiess A. P. Wolchok J. D. Barker C. A. Postow M. A. Tabar V. Huse J. T. (2015). Stereotactic radiosurgery for melanoma brain metastases in patients receiving ipilimumab: safety profile and efficacy of combined treatment. Int. J. Radiat. Oncol. Biol. Phys. 92 (2), 368–375. Epub 2015 Mar 5. PMID: 25754629; PMCID: PMC4955924. 10.1016/j.ijrobp.2015.01.004 25754629 PMC4955924

[B62] Kim T. K. Vandsemb E. N. Herbst R. S. Chen L. (2022). Adaptive immune resistance at the tumour site: mechanisms and therapeutic opportunities. Nat. Rev. Drug Discov. 21 (7), 529–540. 10.1038/s41573-022-00493-5 35701637

[B63] Klapper J. A. Downey S. G. Smith F. O. Yang J. C. Hughes M. S. Kammula U. S. (2008). High-dose interleukin-2 for the treatment of metastatic renal cell carcinoma: a retrospective analysis of response and survival in patients treated in the surgery branch at the National Cancer Institute between 1986 and 2006. Cancer 113 (2), 293–301. 10.1002/cncr.23552 18457330 PMC3486432

[B64] Klarquist J. Hennies C. M. Lehn M. A. Reboulet R. A. Feau S. Janssen E. M. (2014). STING-mediated DNA sensing promotes antitumor and autoimmune responses to dying cells. J. Immunol. 193 (12), 6124–6134. 10.4049/jimmunol.1401869 25385820 PMC4258444

[B65] Klug F. Prakash H. Huber P. E. Seibel T. Bender N. Halama N. (2013). Low-dose irradiation programs macrophage differentiation to an iNOS^+^/M1 phenotype that orchestrates effective T cell immunotherapy. Cancer Cell 24 (5), 589–602. 10.1016/j.ccr.2013.09.014 24209604

[B66] Kwon E. D. Drake C. G. Scher H. I. Fizazi K. Bossi A. van den Eertwegh A. J. (2014). Ipilimumab versus placebo after radiotherapy in patients with metastatic castration-resistant prostate cancer that had progressed after docetaxel chemotherapy (CA184-043): a multicentre, randomised, double-blind, phase 3 trial. Lancet Oncol. 15 (7), 700–712. 10.1016/S1470-2045(14)70189-5 24831977 PMC4418935

[B67] Lee E. C. Y. Kok J. S. T. Teh B. T. Lim K. S. (2022). Interplay between the DNA damage response and immunotherapy response in cancer. Int. J. Mol. Sci. 23 (21), 13356. 10.3390/ijms232113356 36362142 PMC9654744

[B68] Li G. M. (2008). Mechanisms and functions of DNA mismatch repair. Cell Res. 18 (1), 85–98. 10.1038/cr.2007.115 18157157

[B69] Li T. Chen Z. J. (2018). The cGAS-cGAMP-STING pathway connects DNA damage to inflammation, senescence, and cancer. J. Exp. Med. 215 (5), 1287–1299. 10.1084/jem.20180139 29622565 PMC5940270

[B70] Lim S. M. Peters S. Ortega Granados A. L. Pinto G. D. J. Fuentes C. S. Lo Russo G. (2023). Dostarlimab or pembrolizumab plus chemotherapy in previously untreated metastatic non-squamous non-small cell lung cancer: the randomized PERLA phase II trial. Nat. Commun. 14 (1), 7301. PMID: 37951954; PMCID: PMC10640551. 10.1038/s41467-023-42900-4 37951954 PMC10640551

[B71] Lutsiak M. E. C. Semnani R. T. De Pascalis R. Kashmiri S. V. S. Schlom J. Sabzevari H. (2005). Inhibition of CD4(+)25+ T regulatory cell function implicated in enhanced immune response by low-dose cyclophosphamide. Blood 105 (7), 2862–2868. 10.1182/blood-2004-06-2410 15591121

[B72] Mavragani I. V. Laskaratou D. A. Frey B. Candéias S. M. Gaipl U. S. Lumniczky K. (2015). Key mechanisms involved in ionizing radiation-induced systemic effects. A current review. Toxicol. Res. (Camb). 5 (1), 12–33. 10.1039/c5tx00222b 30090323 PMC6061884

[B73] Maynard S. Schurman S. H. Harboe C. de Souza-Pinto N. C. Bohr V. A. (2009). Base excision repair of oxidative DNA damage and association with cancer and aging. Carcinogenesis 30 (1), 2–10. 10.1093/carcin/bgn250 18978338 PMC2639036

[B74] McGovern K. Ghaly M. Esposito M. Barnaby K. Seetharamu N. (2019). Radiation recall pneumonitis in the setting of immunotherapy and radiation: a focused review. Future Sci. oa. 5:(5), FSO378. 10.2144/fsoa-2018-0123 31245041 PMC6554692

[B75] McLaughlin M. Patin E. C. Pedersen M. Wilkins A. Dillon M. T. Melcher A. A. (2020). Inflammatory microenvironment remodelling by tumour cells after radiotherapy. Nat. Rev. Cancer 20 (4), 203–217. 10.1038/s41568-020-0246-1 32161398

[B76] Mellman I. Coukos G. Dranoff G. (2011). Cancer immunotherapy comes of age. Nature 480 (7378), 480–489. 10.1038/nature10673 22193102 PMC3967235

[B77] Menon H. Chen D. Ramapriyan R. Verma V. Barsoumian H. B. Cushman T. R. (2019). Influence of low-dose radiation on abscopal responses in patients receiving high-dose radiation and immunotherapy. J. Immunother. Cancer 7 (1), 237. 10.1186/s40425-019-0718-6 31484556 PMC6727581

[B78] Morris Z. S. Guy E. I. Francis D. M. Gressett M. M. Werner L. R. Carmichael L. L. (2016). *In situ* tumor vaccination by combining local radiation and tumor-specific antibody or immunocytokine treatments. Cancer Res. 76 (13), 3929–3941. 10.1158/0008-5472.CAN-15-2644 27197149 PMC4930687

[B79] Motwani M. Pesiridis S. Fitzgerald K. A. (2019). DNA sensing by the cGAS-STING pathway in health and disease. Nat. Rev. Genet. 20 (11), 657–674. 10.1038/s41576-019-0151-1 31358977

[B80] Mushtaq A. Mir U. S. Hunt C. R. Pandita S. Tantray W. W. Bhat A. (2021). Role of histone methylation in maintenance of genome integrity. Genes. 12 (7), 1000. 10.3390/genes12071000 34209979 PMC8307007

[B81] Nakahara T. Uchi H. Lesokhin A. M. Avogadri F. Rizzuto G. A. Hirschhorn-Cymerman D. (2010). Cyclophosphamide enhances immunity by modulating the balance of dendritic cell subsets in lymphoid organs. Blood 115 (22), 4384–4392. 10.1182/blood-2009-11-251231 20154220 PMC2881499

[B82] NCBI (2023). The cytosolic DNA sensor cGAS forms an oligomeric complex with DNA and undergoes switch-like conformational changes in the activation loop. Available at: https://pubmed.ncbi.nlm.nih.gov/24462292/(Accessed August 29, 2023). 10.1016/j.celrep.2014.01.003 PMC3969844 24462292

[B83] NCBI (2024a). Histone gammaH2AX and poly(ADP-ribose) as clinical pharmacodynamic biomarkers. Available at: https://pubmed.ncbi.nlm.nih.gov/20823146/. 10.1158/1078-0432.CCR-10-0523 PMC2940983 20823146

[B84] NCBI (2024b). Mechanisms of DNA damage, repair and mutagenesis. Available at: https://www.ncbi.nlm.nih.gov/pmc/articles/PMC5474181/. 10.1002/em.22087 PMC5474181 28485537

[B85] NCBI (2024c). Ubiquitin specific peptidase 37 and PCNA interaction promotes osteosarcoma pathogenesis by modulating replication fork progression. Available at: https://pubmed.ncbi.nlm.nih.gov/37118828/. 10.1186/s12967-023-04126-2 PMC10142227 37118828

[B86] Patel R. R. Verma V. Barsoumian H. B. Ning M. S. Chun S. G. Tang C. (2021). Use of multi-site radiation therapy for systemic disease control. Int. J. Radiat. Oncol. Biol. Phys. 109 (2), 352–364. 10.1016/j.ijrobp.2020.08.025 32798606 PMC10644952

[B87] Peters S. Felip E. Dafni U. Tufman A. Guckenberger M. Álvarez R. (2021). Progression-free and overall survival for concurrent nivolumab with standard concurrent chemoradiotherapy in locally advanced stage IIIA-B NSCLC: results from the European thoracic oncology platform NICOLAS phase II trial (European thoracic oncology platform 6-14). J. Thorac. Oncol. 16 (2), 278–288. 10.1016/j.jtho.2020.10.129 33188912

[B88] Peters S. Gettinger S. Johnson M. L. Jänne P. A. Garassino M. C. Christoph D. (2017). Phase II trial of atezolizumab as first-line or subsequent therapy for patients with programmed death-ligand 1-selected advanced non-small-cell lung cancer (BIRCH). J. Clin. Oncol. 35 (24), 2781–2789. 10.1200/JCO.2016.71.9476 28609226 PMC5562171

[B89] Qin A. Rengan R. Lee S. Santana-Davila R. Goulart B. H. L. Martins R. (2020). A pilot study of atezolizumab plus hypofractionated image guided radiation therapy for the treatment of advanced non-small cell lung cancer. Int. J. Radiat. Oncol. Biol. Phys. 108 (1), 170–177. Epub 2019 Nov 19. PMID: 31756415. 10.1016/j.ijrobp.2019.10.047 31756415

[B90] Reisz J. A. Bansal N. Qian J. Zhao W. Furdui C. M. (2014). Effects of ionizing radiation on biological molecules—mechanisms of damage and emerging methods of detection. Antioxid. Redox Signal 21 (2), 260–292. 10.1089/ars.2013.5489 24382094 PMC4060780

[B91] Rhea L. P. Aragon-Ching J. B. (2021). Advances and controversies with checkpoint inhibitors in bladder cancer. Clin. Med. Insights Oncol. 15, 11795549211044963. PMID: 34602833; PMCID: PMC8481722. 10.1177/11795549211044963 34602833 PMC8481722

[B92] Rodríguez-Ruiz M. E. Vanpouille-Box C. Melero I. Formenti S. C. Demaria S. (2018). Immunological mechanisms responsible for radiation-induced abscopal effect. Trends Immunol. 39 (8), 644–655. 10.1016/j.it.2018.06.001 30001871 PMC6326574

[B93] Saad A. Goldstein J. Appel S. Daher S. Urban D. Onn A. (2022). Chemoradiation followed by adjuvant durvalumab in stage III non-small cell lung cancer: real-world comparison of treatment outcomes to historical controls treated with chemoradiation alone. Thorac. Cancer 13 (12), 1763–1771. Epub 2022 May 11. PMID: 35538909; PMCID: PMC9200887. 10.1111/1759-7714.14452 35538909 PMC9200887

[B94] Said S. S. Ibrahim W. N. (2023). Cancer resistance to immunotherapy: comprehensive insights with future perspectives. Pharmaceutics 15 (4), 1143. 10.3390/pharmaceutics15041143 37111629 PMC10141036

[B95] Saito G. Oya Y. Taniguchi Y. Kawachi H. Daichi F. Matsumoto H. (2021). Real-world survey of pneumonitis and its impact on durvalumab consolidation therapy in patients with non-small cell lung cancer who received chemoradiotherapy after durvalumab approval (HOPE-005/CRIMSON). Lung Cancer. 161, 86–93. 10.1016/j.lungcan.2021.08.019 34543942

[B96] San Filippo J. Sung P. Klein H. (2008). Mechanism of eukaryotic homologous recombination. Annu. Rev. Biochem. 77 (1), 229–257. 10.1146/annurev.biochem.77.061306.125255 18275380

[B97] Sharabi A. B. Nirschl C. J. Kochel C. M. Nirschl T. R. Francica B. J. Velarde E. (2015). Stereotactic radiation therapy augments antigen-specific PD-1-mediated antitumor immune responses via cross-presentation of tumor antigen. Cancer Immunol. Res. 3 (4), 345–355. 10.1158/2326-6066.CIR-14-0196 25527358 PMC4390444

[B98] Sharma P. Hu-Lieskovan S. Wargo J. A. Ribas A. (2017). Primary, adaptive, and acquired resistance to cancer immunotherapy. Cell 168 (4), 707–723. 10.1016/j.cell.2017.01.017 28187290 PMC5391692

[B99] Shaverdian N. Lisberg A. E. Bornazyan K. Veruttipong D. Goldman J. W. Formenti S. C. (2017). Previous radiotherapy and the clinical activity and toxicity of pembrolizumab in the treatment of non-small-cell lung cancer: a secondary analysis of the KEYNOTE-001 phase 1 trial. Lancet Oncol. 18 (7), 895–903. 10.1016/S1470-2045(17)30380-7 28551359 PMC5538772

[B100] Shinohara E. T. Geng L. Tan J. Chen H. Shir Y. Edwards E. (2005). DNA-dependent protein kinase is a molecular target for the development of noncytotoxic radiation-sensitizing drugs. Cancer Res. 65 (12), 4987–4992. 10.1158/0008-5472.CAN-04-4250 15958537

[B101] Shiravand Y. Khodadadi F. Kashani S. M. A. Hosseini-Fard S. R. Hosseini S. Sadeghirad H. (2022). Immune checkpoint inhibitors in cancer therapy. Curr. Oncol. 29 (5), 3044–3060. 10.3390/curroncol29050247 35621637 PMC9139602

[B102] Spigel D. R. Faivre-Finn C. Gray J. E. Vicente D. Planchard D. Paz-Ares L. (2022). Five-year survival outcomes from the PACIFIC trial: durvalumab after chemoradiotherapy in stage III non-small-cell lung cancer. J. Clin. Oncol. 40:(12), 1301–1311. 10.1200/JCO.21.01308 35108059 PMC9015199

[B103] Sprooten J. Agostinis P. Garg A. D. (2019). Type I interferons and dendritic cells in cancer immunotherapy. Int. Rev. Cell Mol. Biol. 348, 217–262. 10.1016/bs.ircmb.2019.06.001 31810554

[B104] Srinivas U. S. Tan B. W. Q. Vellayappan B. A. Jeyasekharan A. D. (2018). ROS and the DNA damage response in cancer. Redox Biol. 25, 101084. 10.1016/j.redox.2018.101084 30612957 PMC6859528

[B105] Strzalka W. Ziemienowicz A. (2011). Proliferating cell nuclear antigen (PCNA): a key factor in DNA replication and cell cycle regulation. Ann. Bot. 107 (7), 1127–1140. 10.1093/aob/mcq243 21169293 PMC3091797

[B106] Sundahl N. Vandekerkhove G. Decaestecker K. Meireson A. De Visschere P. Fonteyne V. (2019). Randomized phase 1 trial of pembrolizumab with sequential versus concomitant stereotactic body radiotherapy in metastatic urothelial carcinoma. Eur. Urol. 75 (5), 707–711. 10.1016/j.eururo.2019.01.009 30665814

[B107] Tang C. Wang X. Soh H. Seyedin S. Cortez M. A. Krishnan S. (2014). Combining radiation and immunotherapy: a new systemic therapy for solid tumors? Cancer Immunol. Res. 2 (9), 831–838. 10.1158/2326-6066.CIR-14-0069 25187273 PMC5367158

[B108] Tang C. Welsh J. W. de Groot P. Massarelli E. Chang J. Y. Hess K. R. (2017). Ipilimumab with stereotactic ablative radiation therapy: phase I results and immunologic correlates from peripheral T cells. Clin. Cancer Res. 23 (6), 1388–1396. 10.1158/1078-0432.CCR-16-1432 27649551 PMC5355002

[B109] Turgeon G. A. Weickhardt A. Azad A. A. Solomon B. Siva S. (2019). Radiotherapy and immunotherapy: a synergistic effect in cancer care. Med. J. Aust. 210 (1), 47–53. 10.5694/mja2.12046 30636308

[B110] Vaddepally R. K. Kharel P. Pandey R. Garje R. Chandra A. B. (2020). Review of indications of FDA-approved immune checkpoint inhibitors per NCCN guidelines with the level of evidence. Cancers (Basel) 12 (3), 738. 10.3390/cancers12030738 32245016 PMC7140028

[B111] Vereecque R. Saudemont A. Quesnel B. (2004). Cytosine arabinoside induces costimulatory molecule expression in acute myeloid leukemia cells. Leukemia 18 (7), 1223–1230. 10.1038/sj.leu.2403391 15152266

[B112] Waldman A. D. Fritz J. M. Lenardo M. J. (2020). A guide to cancer immunotherapy: from T cell basic science to clinical practice. Nat. Rev. Immunol. 20 (11), 651–668. 10.1038/s41577-020-0306-5 32433532 PMC7238960

[B113] Wang J. Ge H. Tian Z. (2023). Immunotherapy plus radiotherapy for the treatment of sarcomas: is there a potential for synergism? Onco Targets Ther. 16, 385–397. 10.2147/ott.s410693 37313391 PMC10258041

[B114] Wang L. Amoozgar Z. Huang J. Saleh M. H. Xing D. Orsulic S. (2015). Decitabine enhances lymphocyte migration and function and synergizes with CTLA-4 blockade in a murine ovarian cancer model. Cancer Immunol. Res. 3 (9), 1030–1041. 10.1158/2326-6066.CIR-15-0073 26056145

[B115] Wang Y. Duan M. Peng Z. Fan R. He Y. Zhang H. (2022). Advances of DNA damage repair-related drugs and combination with immunotherapy in tumor treatment. Front. Immunol. 13, 854730. 10.3389/fimmu.2022.854730 35281059 PMC8904426

[B116] Weber S. (2005). Light-driven enzymatic catalysis of DNA repair: a review of recent biophysical studies on photolyase. Biochim. Biophys. Acta 1707 (1), 1–23. 10.1016/j.bbabio.2004.02.010 15721603

[B117] Weichselbaum R. R. Liang H. Deng L. Fu Y. X. (2017). Radiotherapy and immunotherapy: a beneficial liaison? Nat. Rev. Clin. Oncol. 14 (6), 365–379. 10.1038/nrclinonc.2016.211 28094262

[B118] Yan H. Song L. Li Y. Xu Q. Guo W. Lin S. (2024). Clinical evidence for efficacy of pembrolizumab in MSI-H and TMB-H advanced solid tumor: results from three cancer centers in China. Cancer Immunol. Immunother. 73 (4), 74. PMID: 38451314; PMCID: PMC10920474. 10.1007/s00262-024-03660-2 38451314 PMC10920474

[B119] Ye Z. Shi Y. Lees-Miller S. P. Tainer J. A. (2021). Function and molecular mechanism of the DNA damage response in immunity and cancer immunotherapy. Front. Immunol. 12, 797880. 10.3389/fimmu.2021.797880 34970273 PMC8712645

[B120] Ye Z. M. Tang Z. Q. Xu Z. Zhou Q. Li H. (2022). Cost-effectiveness of nivolumab plus ipilimumab as first-line treatment for American patients with unresectable malignant pleural mesothelioma. Front. Public Health 10, 947375. PMID: 35937220; PMCID: PMC9354521. 10.3389/fpubh.2022.947375 35937220 PMC9354521

[B121] Yu R. Zhu B. Chen D. (2022b). Type I interferon-mediated tumor immunity and its role in immunotherapy. Cell Mol. Life Sci. 79 (3), 191. 10.1007/s00018-022-04219-z 35292881 PMC8924142

[B122] Yu S. Wang Y. He P. Shao B. Liu F. Xiang Z. (2022a). Effective combinations of immunotherapy and radiotherapy for cancer treatment. Front. Oncol. 12, 809304. 10.3389/fonc.2022.809304 35198442 PMC8858950

[B123] Zhai X. Zhang J. Tian Y. Li J. Jing W. Guo H. (2020). The mechanism and risk factors for immune checkpoint inhibitor pneumonitis in non-small cell lung cancer patients. Cancer Biol. Med. 17 (3), 599–611. 10.20892/j.issn.2095-3941.2020.0102 32944393 PMC7476083

[B124] Zhang J. Shih D. J. H. Lin S. Y. (2020). Role of DNA repair defects in predicting immunotherapy response. Biomark. Res. 8 (1), 23. 10.1186/s40364-020-00202-7 32612833 PMC7325270

[B125] Zhang L. Dermawan K. Jin M. Liu R. Zheng H. Xu L. (2008). Differential impairment of regulatory T cells rather than effector T cells by paclitaxel-based chemotherapy. Clin. Immunol. 129 (2), 219–229. 10.1016/j.clim.2008.07.013 18771959

[B126] Zhang Q. Tang L. Zhou Y. He W. Li W. (2021). Immune checkpoint inhibitor-associated pneumonitis in non-small cell lung cancer: current understanding in characteristics, diagnosis, and management. Front. Immunol. 12, 663986. 10.3389/fimmu.2021.663986 34122422 PMC8195248

[B127] Zhu S. Zhang T. Zheng L. Liu H. Song W. Liu D. (2021a). Combination strategies to maximize the benefits of cancer immunotherapy. J. Hematol. Oncol. 14 (1), 156. 10.1186/s13045-021-01164-5 34579759 PMC8475356

[B128] Zhu S. Zhang T. Zheng L. Liu H. Song W. Liu D. (2021b). Combination strategies to maximize the benefits of cancer immunotherapy. J. Hematol. Oncol. 14 (1), 156. 10.1186/s13045-021-01164-5 34579759 PMC8475356

[B129] Zimmer L. Livingstone E. Hassel J. C. Fluck M. Eigentler T. Loquai C. (2020). Adjuvant nivolumab plus ipilimumab or nivolumab monotherapy versus placebo in patients with resected stage IV melanoma with no evidence of disease (IMMUNED): a randomised, double-blind, placebo-controlled, phase 2 trial. Lancet 395 (10236), 1558–1568. 10.1016/S0140-6736(20)30417-7 32416781

